# Interaction of planting system with radiation‐use efficiency in wheat lines

**DOI:** 10.1002/csc2.21115

**Published:** 2023-11-14

**Authors:** Marcela A. Moroyoqui‐Parra, Gemma Molero, Matthew P. Reynolds, Oorbessy Gaju, Erik H. Murchie, Michael John Foulkes

**Affiliations:** ^1^ Division of Plant and Crop Science, School of Biosciences University of Nottingham Leicestershire UK; ^2^ Global Wheat Program International Maize and Wheat Improvement Center (CIMMYT) Texcoco Mexico; ^3^ KWS Momont Recherche Mons‐en‐Pevele France; ^4^ Lincoln Institute for Agri‐Food and Technology University of Lincoln Lincoln UK

## Abstract

Radiation‐use efficiency (RUE) is an important trait for raising biomass and yield potential in plant breeding. However, the effect of the planting system (PS) on genetic variation in RUE has not been previously investigated. Our objectives were to quantify genetic variation in RUE, biomass and grain yield in raised‐bed and flat‐basin planting systems, and associations with canopy‐architecture traits (flag‐leaf angle and curvature). Twelve spring wheat (*Triticum aestivum* L.) cultivars were evaluated under irrigated conditions for 3 years in North West Mexico using raised‐bed and flat‐basin planting systems. Canopy architecture traits were measured at booting and anthesis + 7 days. Grain yield (10.6%), biomass (7.6%), and pre‐grain‐filling RUE (9.7%) were higher in raised beds than flat basins, while a significant planting system × genotype interaction was found for grain yield. Genetic variation in pre‐grain‐filling RUE was associated with biomass and grain yield in beds and basins. In flat basins, higher pre‐grain‐filling RUE was correlated with a more upright flag‐leaf angle but not in raised beds. In raised beds, cultivars with less upright flag‐leaf angle had greater fractional light interception pre‐anthesis. Taller semi‐dwarf cultivars intercepted relatively more radiation in the beds than the flats before anthesis, consistent with the taller cultivars showing relatively greater increases in yield in beds compared to flats. Our results indicated that the evaluation of genotypes for RUE and biomass in wheat breeding should take into account planting systems to capture genotype × PS effects. In addition, the results demonstrate how flag‐leaf angle has a different effect depending on the planting system.

## Core Ideas

- Yield, biomass, and radiation‐use efficiency (RUE) of wheat lines were higher on raised beds than flat basins.
- Yield showed a planting system × genotype interaction; taller cultivars performed relatively better in beds than basins.
- In basins, higher RUE among cultivars was associated with a more upright flag‐leaf angle.
- In beds, higher fractional light interception among cultivars was associated with a less upright flag‐leaf angle.
- Evaluation of RUE, biomass, and other yield‐related traits in wheat breeding should take account of the effects of the planting system.

**Abbreviations**
BMPM: biomass at physiological maturity
FLA: flag‐leaf angle
FLcv: flag‐leaf curvature
FLcvs: flag‐leaf curvature score
PAR: photosynthetically active radiation
PS: planting system
RUE: radiation‐use efficiency

## 1 INTRODUCTION

**FIGURE 1:**
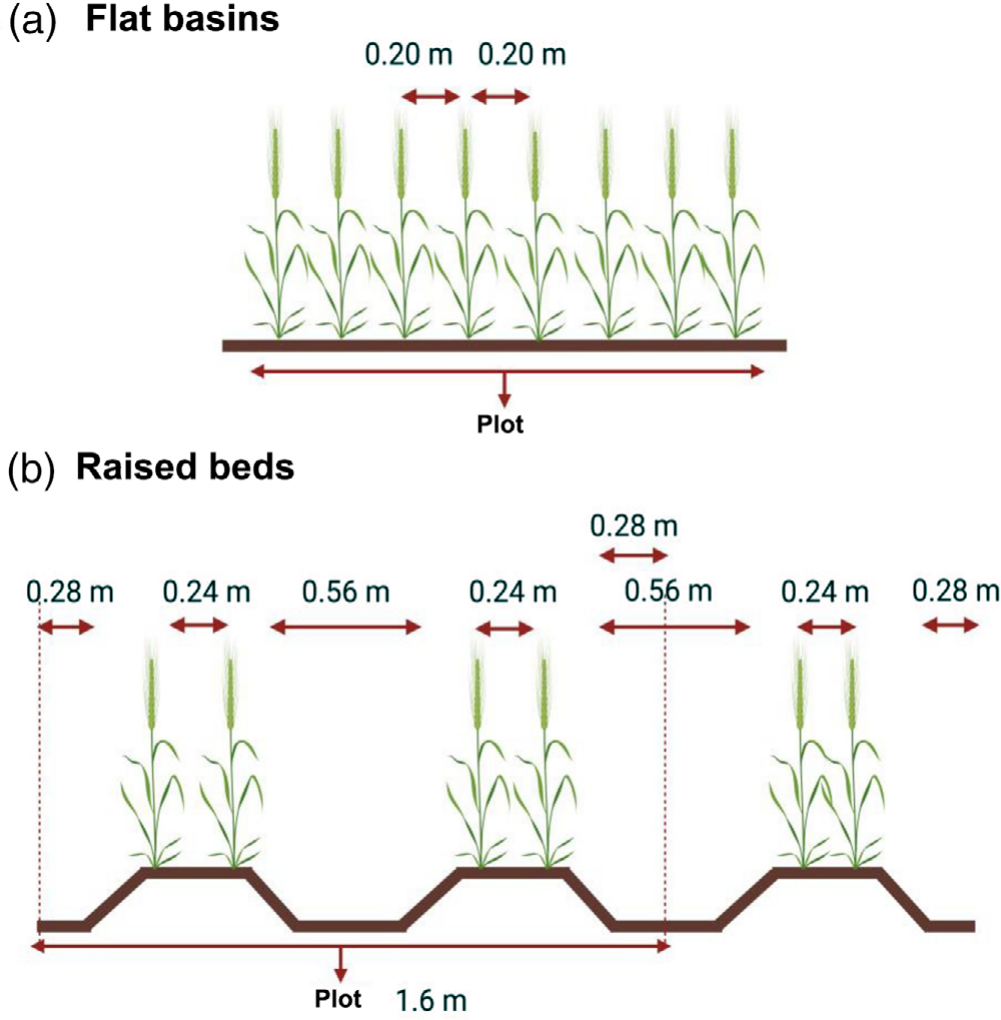
Dimensions for (a) flat basins: same distance between the 8 rows and (b) raised beds: two rows per bed with a wider furrow gap. Adapted from Fischer (2005). Created with Biorender.com.

Wheat (*Triticum aestivum* L.) is the most widely grown crop with ∼750 million tons produced every year (FAOSTAT, 2018) and contributes 20% of the calories of the global human diet (Braun et al., 2010). Wheat yields will need to be doubled by 2050 for food security (Ray et al., 2013). Van Dijk et al. (2021) predicted an increased food demand by 35%−56% between 2010 and 2050. In recent decades in irrigated wheat production in North West Mexico, growers have adopted a raised‐bed planting system (Figure 1) converting from the traditional flat‐basin planting system (Fahong et al., 2004). The raised‐bed planting system consists of defined rows planted on the top of the beds with flood irrigation supplied in furrows between the beds and has been associated with grain yield improvements (Fahong et al., 2004). In addition, management advantages of raised beds compared with flat basins are as follows: reduced irrigation water requirements by 20%–40%, reduced weeds/diseases associated with the wider row spacings facilitating weed control and reducing disease pressure, improved nitrogen (N) fertilizer‐use efficiency, reduced lodging, and improved plant establishment (Sayre et al., 2008). However, the results of investigations comparing raised beds versus flat basins are inconsistent in terms of grain yield effects, and there is a need for further studies. Better grain yield in raised beds than flat basins was reported in wheat by 4%–17% (Ahmad et al., 2010; Fahong et al., 2004; Jat et al., 2011; Kong et al., 2010; Majeed et al., 2015; Noorka & Tabasum, 2013; Wang et al., 2009). However, Tanveer et al. (2003) and López‐Castañeda et al. (2014) found greater grain yield in flat basins than raised beds. Some studies have reported a trend for taller cultivars in raised beds to perform relatively better than in flat basins, potentially associated with earlier canopy closure in the gap between the beds leading to higher radiation interception pre‐anthesis (Fischer, 2005) and/or to effects on radiation‐use efficiency (RUE). Therefore, the better performing cultivars in flat basins may not be better performing in raised beds. Yield potential (YP) can be expressed by simple Equation (1) (Reynolds et al., 2005):

(1) YP=LI×RUE×HI,

where LI is intercepted radiation, RUE is radiation‐use efficiency (ratio of aboveground biomass to radiation intercepted; (Monteith & Moss, 1977), and HI is harvest index (ratio of grain biomass to aboveground biomass).

During the Green Revolution, many studies observed that grain yield increased due to a greater HI without improvements in aboveground biomass (Austin et al., 1980; Gifford et al., 1984; Reynolds et al., 1999; Sayre et al., 1997). Since then, HI has shown slower genetic progress and is approaching its theoretical maximum of ca. 0.65 in some countries (Austin, 1980; Foulkes et al., 2011). In the United Kingdom, genetic gains in grain yield from 1980 to 1995 were associated with aboveground biomass; and biomass gains, in turn, were associated with RUE (Shearman et al., 2005). Other studies (Aisawi et al., 2015; Donmez et al., 2001; Reynolds et al., 1999) also demonstrated genetic progress in aboveground biomass in wheat in the last decades. Wheat canopies in favorable conditions typically achieve >95% light interception from canopy closure at the onset of stem extension up to mid grain‐filling when flag‐leaf senescence begins (Reynolds et al., 2005). Therefore, to raise biomass, RUE must be improved in future breeding since light interception is close to approaching its upper limit (Foulkes & Murchie, 2011).

**Core Ideas**

- Yield, biomass, and radiation‐use efficiency (RUE) of wheat lines were higher on raised beds than flat basins.
- Yield showed a planting system × genotype interaction; taller cultivars performed relatively better in beds than basins.
- In basins, higher RUE among cultivars was associated with a more upright flag‐leaf angle.
- In beds, higher fractional light interception among cultivars was associated with a less upright flag‐leaf angle.
- Evaluation of RUE, biomass, and other yield‐related traits in wheat breeding should take account of the effects of the planting system.

In wheat crops, RUE is typically in the region of 2.8 g MJ^−1^ of photosynthetically active radiation (PAR, 400–700 nm) (Molero et al., 2019; Yunusa et al., 1993) and 1.4 g MJ^−1^ of solar radiation (Sinclair & Muchow, 1999). Several recent investigations have demonstrated that RUE has been associated with genetic progress in biomass improvement in bread wheat (e.g. Molero et al., 2019). Reynolds and Pfeiffer (2000) and Shearman et al. (2005) proposed that optimized source‐sink balance might be one feasible approach to enhance RUE. Indeed, crosses between high source lines and lines favoring sink variables such as HI, grain number, and thousand‐grain weight (TGW) in wheat have achieved gains in RUE in the progeny (Reynolds et al., 2017). Alternatively, differences in canopy architecture traits among genotypes may affect RUE. Recent studies in spring CIMMYT wheat cultivars have found a trade‐off between biomass HI and biomass at physiological maturity, so it will be also important to consider strategies to maintain HI in new cultivars with increased biomass (Aisawi et al., 2015; Rivera‐Amado et al., 2019).

More upright leaves have been related with a greater RUE through reduced light saturation of photosynthesis of upper leaves (Duncan, 1971; Isidro et al., 2012). Excessive radiation intercepted by the upper leaf layers causes photooxidative damage to the photosynthesis apparatus (PSII reaction center) (Parry et al., 2011), which can be reduced by more erect leaves (Murchie et al., 1999). The concept of an ideal plant architecture “a smart canopy” in a cereal crop with an erect leaf angle at the top of the canopy, less upright leaves in the middle of the canopy, and more planophiles leaves in the lower canopy has also been proposed (Duncan, 1971; Ku et al., 2010; Mantilla‐Perez et al., 2020; Ort et al., 2015; Zhu et al., 2010). In a recent study in spring wheat lines in Australia, erect canopies yielded 13% more than canopies with lax leaves (Richards et al., 2019) associated with greater biomass. Several other studies reported the advantages of erectophile lines compared to planophile lines, for example in maize (*Zea mays* L.) (Cabrera‐Bosquet et al., 2016; Duncan, 1971; Ku et al., 2010), rice (*Oryza sativa*) (Horton, 2000; Murchie et al., 1999; Peng et al., 2008), and wheat (Austin et al., 1976; Innes & Blackwell, 2009; Reynolds et al., 2012; Townsend et al., 2018; Yang et al., 2016). However, to date, there are no studies comparing the quantitative relationship between canopy architecture traits and RUE among genotypes in raised‐bed and flat‐basin planting systems and the associated effects on biomass and grain yield. It is important to highlight that bread spring wheat germplasm released by CIMMYT, mainly selected under raised‐bed systems, is typically planophile, and although cultivars have been released with a more erect canopy, these are exceptions (Richards et al., 2019). However, bread winter wheats in Europe, selected under flat‐basin systems, tend to be erectophiles. This indicates that canopy architecture could play an important role across planting systems.

In the present study, 12 CIMMYT elite spring wheat cultivars were grown in the raised‐bed and flat‐basin planting systems for 3 years in North West Mexico under irrigated conditions. The aims of this study are (i) to quantify genetic variation in canopy architecture traits and RUE and associations with aboveground biomass and grain yield in the two planting systems and (ii) to understand the physiological basis of the planting system × genotype interaction (PS × G interaction) for grain yield and biomass in relation to the effects of RUE and canopy architecture traits.

## 2 MATERIALS AND METHODS

### 2.1 Experiment site, design, and treatments

Three field experiments were performed at the CIMMYT CENEB (Campo Experimental Norman E. Borlaug) research station in the Yaqui Valley near Ciudad Obregon, Sonora (27°395 N, 109°926 W, 38 masl), under irrigated conditions in 2017–2018, 2018–2019, and 2019–2020. The soil type was a sandy clay, mixed montmorillonitic typic caliciorthid, low in organic matter, and slightly alkaline (pH 7.7) (Sayre et al., 1997). In each year, for each of two planting systems (raised beds and flat basins), a randomized block design was implemented with three replicates per cultivar. The two planting systems were sown in adjacent areas in the field, with a 5‐m gap between the border plots of the planting systems. Ten genotypes were selected based on contrasting RUE, biomass, and canopy architecture from the HiBAP I (High Biomass Association Panel) from previous CIMMYT datasets (Table 1; Molero et al., 2019) and were grown in all three seasons. Two more erectophile cultivars were added in the experiments in 2018–2019 and 2019–2020 to increase canopy architecture variation, selected from the ESWYT (Elite Selection Wheat Yield Trial series) in the CIMMYT wheat breeding program. The genotype names are abbreviated in tables and figures; the full names are given in Table 1 along with information on canopy architecture. Of the 12 genotypes, six were elite CIMMYT cultivars (BACANORA T88, CHEWINK #1, SOKOLL, KUTZ, NELOKI, and BORLAUG100), and the others were elite advanced lines as indicated in Table 1. For concision, the 12 genotypes are referred to as cultivars hereafter.

**TABLE 1:** List of 12 CIMMYT elite spring bread wheat cultivars and advanced lines in the experiments in 2017–2018, 2018–2019, and 2019–2020.

#	Genotype	Canopy architecture type
1	BACANORA T88	Erectophile
2	C80.1/3*QT4118//KAUZ/RAYON/3/2*TRCH/7/CMH79A.955/4/AGA/3/4*SN64/CNO67//INIA66/5/NAC/6/RIALTO	Planophile
3	CHEWINK #1	Planophile
4	SOKOLL//PUB94.15.1.12/WBLL1	Planophile
5	NELOKI	Erectophile
6	W15.92/4/PASTOR//HXL7573/2*BAU/3/WBLL1	Planophile
7	KUKRI	Planophile
8	KUTZ	Planophile
9	SOKOLL	Planophile
10	BORLAUG100 F2014	Planophile
11^a^	ITP40/AKURI//FRNCLN*2/TECUE #1	Erectophile
12^a^	CHIPAK*2//SUP152/KENYA SUNBIRD	Erectophile

^a^ Two lines added in 2018–2019 and 2019–2020.

The raised beds (B) planting system consisted of two beds per plot, whereas the flat basins (F) had one flat area per plot. In the raised beds planting system, the two beds per plot were each 0.8 m wide and 4 m long (=6.4 m^2^ per plot) with two rows per bed (0.24 m between rows) and 0.56 m between the inner rows of the two adjacent beds. Therefore, the plot width in raised beds was 1.6 m (see Figure 1b). For flat basins in 2017–2018 and 2018–2019, there were eight rows per basin (1.6 × 5 m = 8.0 m^2^ per plot) with 0.20 m between rows. In 2019–2020, there were six rows per basin (1.44 × 5 m = 7.2 m^2^ per plot) with 0.24 m between rows. The sowing dates were November 30, 2017, November 30, 2018, and December 21, 2019, for raised beds and December 1, 2017, December 1, 2018, and December 17, 2019, for flat basins. The emergence dates were December 7, 2017, December 7, 2018, and December 31, 2019, in beds and December 9, 2017, December 9, 2018, and December 26, 2020, in flats. The seed rate was 102 kg ha^−1^ for the 3 years in beds and 107 kg ha^−1^ in 2017–2018 and 2018–2019 and 106 kg ha^−1^ in 2019–2020 in flats. In 2017–2018, both planting systems were fertilized with 50 kg N ha^−1^ (urea) during land preparation followed by 50 kg P ha^−1^ at sowing. A second and third N application (200 and 50 kg N ha^−1^, respectively, as urea) was applied at the first and second irrigation, respectively. In 2018–2019 and 2019–2020, both planting systems were fertilized with 50 kg N ha^−1^ (as urea) during land preparation followed by 50 kg P ha^−1^ at sowing. The second N application was added at the time of the first irrigation (200 kg N ha^−1^). In 2017–2018 and 2018–2019, the irrigation was applied every 3–4 weeks during the cycle as flood irrigation in both planting systems. In 2019–2020, raised beds were irrigated as for the first two years, and the flat basins were irrigated using drip irrigation every 3–4 weeks. Herbicides, fungicides, and pesticides were applied as necessary in order to minimize the effects of weeds, diseases, and pests. Plot management information for the two planting systems is summarized in Table S1. In all experiments, there was no evidence for hypoxia in the plants at any stage during the season in any of the plots.

### 2.2 Crop measurements

#### 2.2.1 Phenology and growth analysis

Dates of reaching initiation of booting (GS41), heading (GS55), anthesis (GS65), and physiological maturity (GS87) were recorded (Zadoks et al., 1974) as when 50% of the shoots in the plot reached the stage (Pask et al., 2012). Biomass samples were taken by cutting shoots at the ground level at 40 days after emergence, early booting (GS41), and anthesis (GS65) + 7 days in a 0.8‐m^2^ area (0.50 × 1.6 m) in beds and a 0.8‐m^2^ area (0.50 × 1.6 m) in flats, except for 40 days after emergence (0.40 m^2^: 0.25 × 1.6 m in beds; 0.40 m^2^: 0.25 × 1.6 m in flats). The biomass sample was taken leaving 25 cm (40 days after emergence) or 50 cm (GS41 and GS65 + 7 days) from the end of the plot to reduce border effects. At emergence + 40 days, GS41, and GS65 + 7 days, a subsample of the sampled material was taken on a fresh weight basis of 50 shoots and weighed after drying at 70°C for 48 h. For biomass at physiological maturity, 50 fertile shoots, that is those with a spike (2017–2018 and 2018–2019), or 30 fertile shoots (2019–2020) were sampled randomly by cutting at the ground level to estimate harvest index (HI) (ratio of grain dry matter to aboveground dry matter) and grain yield components as described by Pask et al. (2012). After physiological maturity, grain yield was measured in each plot by machine harvesting an average plot area of 3.2 and 4.0 m^2^ per plot in beds and flats, respectively, and values were further adjusted to the moisture percentage measured in each plot. In each plot, 50 cm at each end of the plot was discarded in order to remove the border effect. For TGW, a subsample of ca. 20 g was taken, and the dry weight was recorded after drying at 70°C for 48 h. The grains were counted using the digital image system Seed Counter (SeedCount SC5000).

Relative chlorophyll content of the flag leaf was measured using a hand‐held SPAD meter (SPAD 502 Minolta) in the middle of the leaf at 7 days after anthesis taking readings for six flag leaves per plot.

#### 2.2.2 Phenology and growth analysis

Fractional interception (FI) of photosynthetically active radiation (PAR, 400–700 nm) was measured using a 1‐m linear ceptometer (AccuPAR LP‐80; Decagon Devices) at emergence + 40 days, GS41, and GS65 + 7 days in 2018–2019 and 2019–2020. The measurements were taken above the crop (incident radiation), inverting the ceptometer 10 cm above the canopy (reflected radiation) and below the canopy at the ground level (transmitted radiation) during sunny days from 11 a.m. to 1 p.m. when the sun was near its zenith and wind speed was low. In the raised beds, the ceptometer was positioned at a certain angle to the direction of the plant rows so that half of the central gap between the beds was included in the measurement to ensure that all variations across the plot were equally sampled, and measurements were representative of the whole plot. A single reading per bed was taken in beds and two readings per plot in flats.

Fractional PAR interception was calculated as follows (Equation 2):

(2) $$\mathrm{FI}=\frac{\left(\mathrm{PARi}-\mathrm{PARr}-\mathrm{PARt}\right)}{\left(\mathrm{PARi}-\mathrm{PARr}\right)}\;,$$

where FI is the fractional PAR interception, PARi is the incident photosynthetically active radiation, PARr is the reflected PAR, and PARt is the transmitted PAR at the soil surface.

#### 2.2.3 Radiation‐use efficiency

RUE was calculated in each plot as the increment in the aboveground dry matter divided by the increment in intercepted PAR (IPAR) for the phase (Monteith & Moss, 1977) in 2018–2019 and 2019–2020. PAR interception was measured at emergence + 40 days, initiation of booting, and anthesis + 7 days to calculate the accumulated PAR during the phenophases: IPARaccE40–InB40 (IPAR accumulated from emergence + 40 days to the initiation of booting), IPARaccInB–A7 (IPAR accumulated from initiation of booting to anthesis + 7 days), IPARaccE40–A7 (IPAR accumulated from emergence + 40 days to anthesis + 7 days), and IPARaccA7–PM (IPAR accumulated from anthesis + 7 days to physiological maturity). The accumulated PAR interception for the phenophases up to GS65 + 7 days was calculated from the cumulative incident PAR for the phase and then multiplied by the average FI from the start to the end of the phase. For PAR accumulated interception from GS65 + 7 days to physiological maturity (IPARaccA7–PM), the FI at GS65 + 7 days was applied to the incident PAR for each day during the phase; the daily increments of PAR interception were then accumulated for the phase (a correction factor of 0.5 was applied to FI during the last 25% of the grain‐filling period to account for the interception by senesced canopy; Reynolds & Pfeiffer, 2000). A PAR interception from emergence + 40 days to physiological maturity was calculated as the sum of IPARaccE40–A7 and IPARaccA7–PM. RUE was measured over five different phases: RUE_E40InB (from 40 days after emergence [close to canopy closure] to the initiation of booting), RUE_InBA7 (from initiation of booting to 7 days after anthesis), RUE_preGF (RUE pre‐grain‐filling from 40 days after emergence to 7 days after anthesis), RUE_GF (RUE grain‐filling from 7 days after anthesis to physiological maturity) and RUET (from emergence + 40 days to physiological maturity). RUE was therefore calculated using Equations ((3), (4), (5), (6), (7)):

(3) $$\mathrm{RUE}\_E40\mathrm{InB}=\frac{\mathrm{BMInB}-\mathrm{BME}40}{\;\mathrm{IPARaccInB}-E40},$$

(4) $$\mathrm{RUE}\_\mathrm{InBA}7=\frac{\mathrm{BMA}7-\mathrm{BMInB}}{\mathrm{IPARaccA}7-\mathrm{InB}},\;$$

(5) $$\mathrm{RUE}\_\mathrm{preGF}=\frac{\mathrm{BMA}7-\mathrm{BME}40}{\;\mathrm{IPARaccA}7-E40},$$

(6) $$\mathrm{RUE}\_\mathrm{GF}=\frac{\mathrm{BMPM}\;-\;\mathrm{BMA}7}{\mathrm{IPARaccPM}-A7\;},$$

(7) $$\mathrm{RUET}=\frac{\mathrm{BMPM}\;-\;\mathrm{BME}40}{\mathrm{IPARaccPM}-E40\;},$$

where BM is the aboveground dry matter, IPARacc is the accumulated intercepted PAR, E40 is emergence + 40 days, InB is initiation of booting (GS41), A7 is the anthesis (GS65) + 7 days, and PM is the physiological maturity.

#### 2.2.4 Canopy architecture traits

**FIGURE 2:**
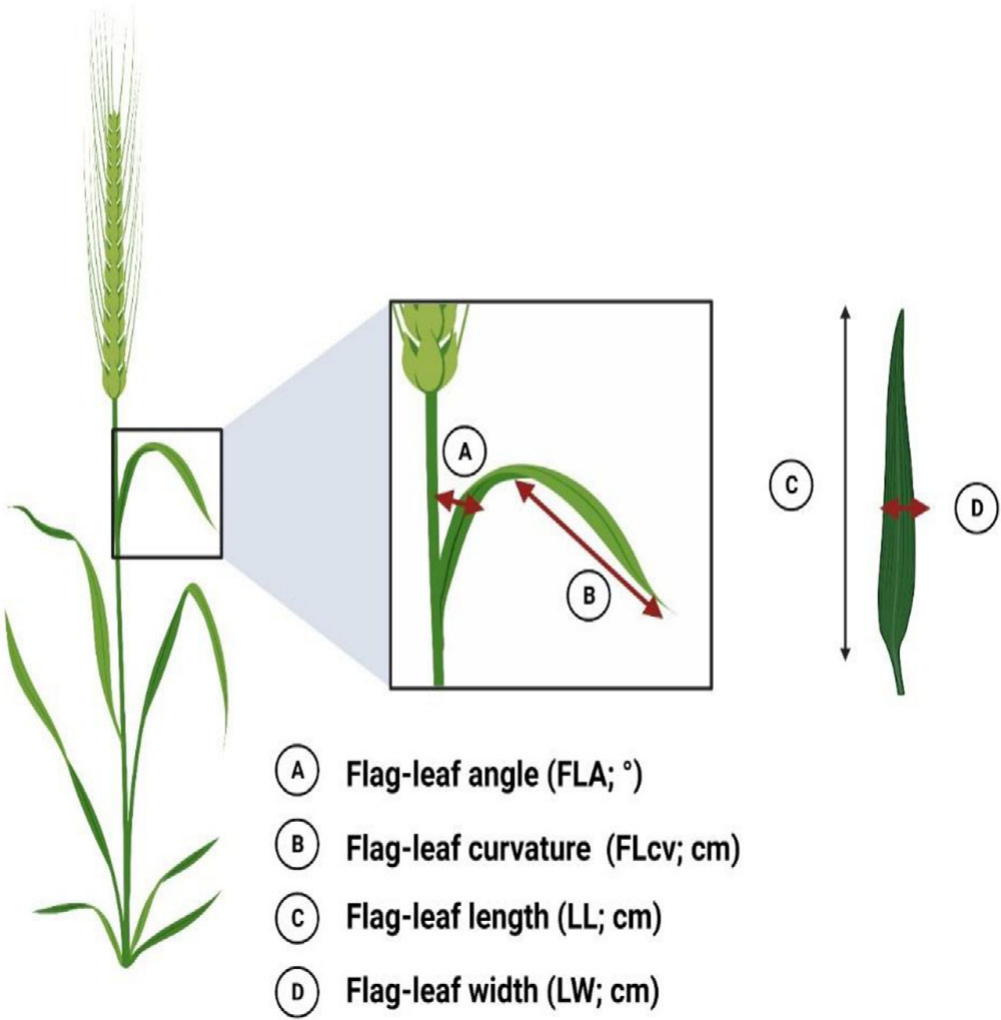
Diagram of canopy architecture measurements on the flag leaf in wheat. Created with Biorender.com.

Canopy architecture was scored for each plot at the initiation of booting and 7 days after anthesis. A qualitative method (visual score) of overall canopy architecture based on the upper leaves (Flag‐leaf canopy visual score; FLcvs) was made for each plot using the scale of Richards et al. (2019). A score of 1 was given to plots when all leaves were visually erect, a score of 6 was given when 60% of the leaves in the upper canopy appeared planophile, and a score of 10 was given to plots when all visible leaves in the upper canopy appeared planophile. More detailed canopy architecture measurements using quantitative methods were taken on the flag leaf for six randomly selected fertile shoots per plot (Figure 2). The flag‐leaf angle (FLA; °) was measured from the vertical stem to the middle part of the flag‐leaf using a protractor. The flag‐leaf curvature (FLcv; cm) was measured as the distance from the point of inflection to the tip of the leaf. Total length and total width of the flag leaf were also measured using a ruler. Plant height was measured from the ground to the tip of the spike (awns were not considered) shortly before physiological maturity.

#### 2.2.5 Normalized difference vegetation index

Normalized difference vegetation index (NDVI) was measured in raised beds and flat basins in each plot from canopy closure (close to onset of stem extension) to late grain filling using a Green Seeker spectroradiometer (Trimble Agriculture) approximately every 2 weeks (Pask et al., 2012). The NDVI measurements provided additional information on green canopy area and senescence profiles to help interpret treatment effects on radiation interception. The spectroradiometer sensor was held 60–120 cm above the crop. NDVI was calculated from measurements of reflectance in the red (680 nm) and near infrared (800 nm) regions of the spectrum using Equation (8):

(8) $$\mathrm{NDVI}=\frac{\left(R800-R\;680\right)}{\left(R800+R680\right)},$$

where R680 and R800 are the reflectance at 680 and 800 nm, respectively.

#### 2.2.6 Statistical analysis

Adjusted means for grain yield, yield components, and physiological traits were calculated using a general linear model analysis of variance procedure from META R 6.04 (Alvarado et al., 2020). Replications, years, and planting systems were considered random effects, and genotypes as a fixed effect. A covariate for anthesis date was used as a fixed effect and was included when significant. Phenotypic correlations between traits were Pearson's correlation coefficient calculated using either the 3‐year genotype means or the 2‐year genotype means. Linear regression analysis was applied to 2‐year or 3‐year genotype means for selected traits. Broad sense heritability (*H* ^2^) was calculated using data across the 3 or 2 years using Equation (9):

(9) $$\;H^{2}=\frac{\sigma_{g}^{2}}{\sigma_{g}^{2}+\frac{\sigma_{gy}^{2}}{y}+\frac{\sigma_{gs\;}^{2}}{s}+\frac{(\sigma_{gy)\left(s\right)}^{2}}{ys}+\frac{\sigma_{e}^{2}}{rys}}\;,$$

where *σ* ^2^ is the error variance, $\sigma_{g}^{2}$ is the genotypic variance, $\sigma_{gy}^{2}$ is the G × Y variance, $\sigma_{gs}^{2}$ is the PS variance, *s* is the number of PS, *y* is the number of years, $\sigma_{e}^{2}$ is the residual variance, and *r* is the number of replicates.

## 3 RESULTS

Meteorological data including mean monthly temperature, relative humidity, rainfall, and solar radiation were collected from a weather station within 1 km of the field experiments. The environmental conditions in the field experiments for the three crop cycles are shown in Figure S1. The mean temperature from December to April was similar in 2018–2019 (18.0°C) and 2019–2020 (17.7°C), but in 2017–2018, it was ca. 1°C warmer (19.1°C). Low rainfall (<10 mm per month) was observed during each of the three crop cycles. Average radiation from December to April was higher during 2017–2018 than in 2018–2019 and 2019–2020.

### 3.1 Gran yield, yield components, and developmental stages

Plant establishment (plant counts in an area of 0.40 m^2^ in beds and 0.40 m^2^ in flats 28 days after emergence) was measured only in 2019–2020 when a nonsignificant difference (153–176 plants m^−2^) was observed between the two planting systems (Table 2).

**TABLE 2:** Mean, minimum, maximum, and analysis of variance (ANOVA) for yield, yield components, biomass at maturity, and phenology expressed in days after emergence (DAE) from the combined analysis across 2017–2018, 2018–2019, and 2019–2020 in raised beds (B) and flat basins (F).

	*p* **value**									
	Mean		Min		Max					
Trait	B	F	B	F	B	F	G	Y	PS	PS × G
YLD (g m^−2^)	666	602	539	525	741	693	<0.001	<0.05	<0.001	<0.05
TGW (g)	45.2	44.8	35.2	35.2	51.2	51.6	<0.001	<0.001	**0.097**	ns
GM2 (m^−2^)	14,883	13,581	11,584	10,611	18,067	16,785	<0.001	<0.01	<0.001	0.184
BMPM (g m^−2^)	1420	1320	1192	1204	1512	1444	<0.001	<0.01	<0.001	<0.05
HeightPM (cm)	105.7	105.7	90.1	88.3	121.3	119.4	<0.001	<0.001	ns	<0.05
ShootsA7 (m^−2^)	451	483	396	395	496	483	<0.001	<0.001	<0.001	<0.05
DTA (DAE)	77	76	72	72	81	80	<0.001	<0.001	<0.001	0.084
DTPM (DAE)	117	114	113	110	121	118	<0.001	<0.001	<0.001	ns
Plants (m^−2^)^a^	156	173	139	152	180	201	<0.001	–	0.074	ns

Abbreviations: BMPM, biomass at physiological maturity; DTA (DAE), days to anthesis (GS65); DTPM (DAE), days to physiological maturity (GS87); GM2, grain number per square meter; HeightPM, plant height at physiological maturity; Min, minimum; Max, maximum; ns, not significant; Plants, number of plants per square meter; ShootsA7, fertile shoots at 7 days to anthesis; TGW, thousand‐grain weight; YLD, grain yield.

^a^ Only 1 year data (2019–2020).

Plant height differed among cultivars from 90.1 to 121.3 cm in beds and from 88.3 to 119.4 cm in flats (*p* < 0.001) with a PS × G interaction (*p* < 0.05), but plant height did not differ between planting systems. The date of the initiation of booting, heading, and anthesis was 1 day later in beds than flats, and for physiological maturity, the date was 3 days later in beds than flats. Averaging across the 3 years, grain yield varied among the cultivars from 539 to 741 g m^−2^ in beds and from 525 to 693 g m^−2^ in flats (*p* < 0.001; Table 2). On average, grain yield in beds was 10.6% higher than in flats (*p* < 0.001). A significant PS × G interaction was observed for yield (*p* < 0.05) with the increase in beds compared to flats ranging from 0 to 120 g m^−2^ among the cultivars. There was a larger grain yield increase in beds compared to flats for taller than shorter cultivars (Table S2). HI was increased in beds (0.47) compared to flats (0.46) (*p* < 0.05), but there was no significant PS × G interaction. Overall, biomass at physiological maturity (BMPM) was 7.6% greater in beds (1420 g m^−2^) than in flats (1320 g m^−2^) (*p* < 0.001). Cultivars ranged from 1192 to 1512 g m^−2^ in beds and from 1204 to 1444 in flats (*p* < 0.001), and there was a significant PS × G interaction. Genetic variation was found for spikes per m^2^, grains per spike (GPS), grains per m^2^ (GM2), and TGW (*p* < 0.001) with a significant planting system effect for all these traits (*p* < 0.01, *p* < 0.001, *p* < 0.001, and *p* = 0.097, respectively). However, only GPS (*p* < 0.001) and SM2 (*p* = 0.068) showed a PS × G interaction. Grain yield was strongly positively correlated with BMPM in beds (*r* = 0.77, *p* < 0.01) and flats (*r* = 0.78, *p* < 0.01; Table 3). A positive correlation was also found between grain yield and HI in flats (*r* = 0.70, *p* < 0.01), but there was no correlation in beds. Results showed that shorter plants had higher HI (B: *r* = −0.53, *p* = 0.08 and F: *r* = −0.62, *p* < 0.05). A strong positive correlation was found between grain yield and GPS in beds (*r* = 0.80, *p* < 0.01) and flats (*r* = 0.61, *p* < 0.05). There was a trade‐off between GM2 and TGW in both planting systems (B: *r* = −0.74, *p* < 0.01, F: *r* = −0.79, *p* < 0.01). Additionally, there was a positive correlation between the anthesis date and BMPM in beds (*r* = 0.58, *p* < 0.05) and flats (*r* = 0.59, *p* < 0.05).

**TABLE 3:** Phenotypic correlations for grain yield, yield components, height at physiological maturity, number of shoots at anthesis + 7 days (m^2^), and phenology in days after emergence (DAE) among the 12 spring CIMMYT wheat genotypes.

	1	2	3	4	5	6	7	8	9
**Raised beds (B)**									
1. YLD	–								
2. TGW	0.42	–							
3. GM2	0.30	−0.74^**^	–						
4. BMPM	0.77^**^	0.70^*^	−0.18	–					
5. SM2	−0.52^****^	−0.87^***^	0.50	−0.71^*^	–				
6. HeightPM	0.34	0.80^**^	−0.61^*^	0.75^**^	−0.81^**^	–			
7. ShootsA7	0.12	−0.47	0.59^*^	−0.11	0.51^****^	−0.55^****^			
8. DTA	0.51^****^	0.16	0.17	0.58^*^	−0.40	0.61^*^	0.96^***^	–	
9. DTPM	0.31	0.03	0.15	0.31	−0.22	0.42	0.81^**^	0.83^***^	–
**Flat basins (F)**									
1. YLD	–								
2. TGW	0.08	–							
3. GM2	0.54^****^	−0.79^**^	–						
4. BMPM	0.78^**^	0.38	0.12	–					
5. SM2	−0.01	−0.84^***^	0.69^*^	−0.37	–				
6. HeightPM	−0.08	0.81^**^	−0.76^**^	0.44	−0.87^***^	–			
7. ShootsA7	0.21	−0.80^**^	0.80^**^	−0.17	0.85^***^	−0.79^**^			
8. DTA	0.25	0.07	0.04	0.59^*^	−0.47	0.56^****^	0.95^***^	–	
9. DTPM	0.16	−0.03	0.08	0.49	−0.35	0.40	0.78^**^	0.81^***^	–

*Note*: Values are based on means in 2017–2018, 2018–2019, and 2019–2020 in raised beds (B) and flat basins (F).

Abbreviations: BMPM, biomass at physiological maturity; DTA (DAE), days to anthesis (GS65); DTPM (DAE), days to physiological maturity (GS87); GM2, grain number per square meter; HeightPM, plant height at physiological maturity; ShootsA7, fertile shoots at 7 days to anthesis; TGW, thousand‐grain weight; YLD, grain yield.

* *p* < 0.05.

** *p* < 0.01.

*** *p* < 0.001.

**** *p* < 0.10.

### 3.2 Canopy architecture traits

Flag‐leaf angle was higher (i.e., less upright leaf) in flat basins (13°) than raised beds (6°) at the initiation of booting (*p* < 0.001). However, at 7 days after anthesis, flag‐leaf angle was higher in raised beds (72°) than flat basins (68°; *p* < 0.05). At the initiation of booting, the flag‐leaf angle varied from 3° (BACANORA T88) to 9° (KUTZ) in raised beds and 4° (BACANORA T88) to 26° (KUTZ) in flat basins (*p* < 0.001) (Table 4). At 7 days after anthesis, flag‐leaf angle varied from 35° (BACANORA T88) to 103° (KUTZ) in raised beds and from 28° to 96° (KUTZ and C80.1/3*QT4118) in flat basins (*p* < 0.001). A PS × G interaction was found for flag‐leaf angle at both stages (*p* < 0.001). The range of the responses to PS (i.e., increase in beds versus flats) for the flag‐leaf angle was from −18° to −1° at GS41 and −15° to 29° at GS65 + 7 days. A planting system effect was observed only for flag‐leaf curvature (FLcv) at the initiation of booting (*p* < 0.01) with higher values in raised beds (higher values, i.e., increased length from inflection point to leaf tip, representing more curved leaf). No planting system effect on the visual score for curvature (FLcvs) was found at either stage. For the quantitative measurement of FLcv, cultivars ranged from 16.4 to 21.8 cm in raised beds and from 6.0 to 17.4 cm in flat basins at the initiation of booting (*p* < 0.001). At 7 days after anthesis, FLcv varied from 8.6 to 23.2 cm in raised beds and from 5.8 to 18.2 cm in flat basins (*p* < 0.001). FLcvs showed genetic variation at GS41 (*p* < 0.001) and GS65 + 7 days (*p* < 0.001). The interaction for FLcv at GS41 ranged from 2.64 to 12.19 cm (increase in beds vs. flats). There was no PS × G interaction for FLcv at GS65 + 7 days, or for FLcvs at either stage. A correlation among cultivars between FLcv and FLcvs was found only in raised beds at GS41 (*r* = 0.51, *p* < 0.01). Flag‐leaf length was higher in flat basins than raised beds at the initiation of booting (*p* < 0.001) but higher in raised beds at 7 days after anthesis (*p* < 0.001) (Table S3).

**TABLE 4:** Flag‐leaf angle (FLA), flag‐leaf curvature visual score (1–10, FLcvs), and distance from the point of inflection of the flag‐leaf to the tip (FLcv) at the initiation of booting (GS41, InB) and 7 days after anthesis (GS65, A7) for 12 CIMMYT spring wheat genotypes from the combined analysis across 2018–2019 and 2019–2020 in raised beds (B) and flat basins (F).

	Initiation of booting						Anthesis + 7 days					
	FLAInB (°)		FLcvInB (cm)		FLcvsInB		FLAA7 (°)		FLcvA7 (cm)		FLcvsA7	
Genotype	B	F	B	F	B	F	B	F	B	F	B	F
BACANORA T88	3	4	22.08	13.86	1	2	35	28	23.64	17.79	3	2
C80.1/3*QT4118	7	20	17.53	12.18	8	6	97	96	17.11	11.33	7	7
CHEWINK#1	7	14	20.97	15.21	9	8	66	61	20.83	15.40	9	8
SOKOLL//PUB94	7	25	17.65	7.91	8	8	53	53	19.51	16.13	7	7
NELOKI	3	6	21.13	16.34	3	3	46	45	20.03	18.52	5	4
W15.92/4/PASTOR	7	12	16.71	7.06	8	7	76	73	18.22	13.37	7	7
KUKRI	8	11	16.38	15.75	8	8	86	98	16.71	6.76	7	7
KUTZ	9	26	18.22	6.03	8	8	103	96	8.59	5.82	7	6
SOKOLL	7	10	17.79	15.15	8	7	67	82	16.59	6.39	7	7
BOURLAG100	8	14	20.18	15.07	9	9	97	68	18.88	12.67	8	8
ITP40/AKURI	4	6	22.33	15.79	2	2	46	49	21.93	17.70	5	2
CHIPAK*2//	6	8	20.78	17.41	5	5	87	63	20.30	8.83	5	5
Mean	6	13	19.31	13.15	6	6	72	68	18.52	12.56	6	6
*H* ^2^		0.99		0.81		0.98		0.99		0.94		0.98
LSD (G) (5%)		2.129		4.026		1.610		10.140		3.829		1.088
CV%		13.53		15.29		16.11		8.98		15.18		10.97
G (*p*‐value)		^***^		^***^		^***^		^***^		^***^		^***^
PS (*p*‐value)		^***^		^**^		0.165		^*^		0.144		0.08
Y (*p*‐value)		0.110		^***^		ns		^*^		^***^		ns
PS × G (*p*‐value)		^***^		0.116		ns		^***^		ns		^***^

Abbreviations: CV, coefficient of variation; FLAInB, flag‐leaf angle at initiation of booting (°); FLcvInB, point of inflection of the flag leaf to the tip (cm); FLcvsInB, flag‐leaf curvature score at initiation of booting; FLAA7, flag‐leaf angle 7 days after anthesis (°); FLcvA7, flag‐leaf curvature at 7 days after anthesis (cm); FLcvsA7score, flag‐leaf curvature score at 7 days after anthesis; LSD, least signficant difference of the means, p = 0.05; ns, not significant; PS × G, planting system × genotype.

* *p* < 0.05.

** *p* < 0.01.

*** *p* < 0.001.

### 3.3 Biomass, radiation interception, and NDVI during the season

**FIGURE 3:**
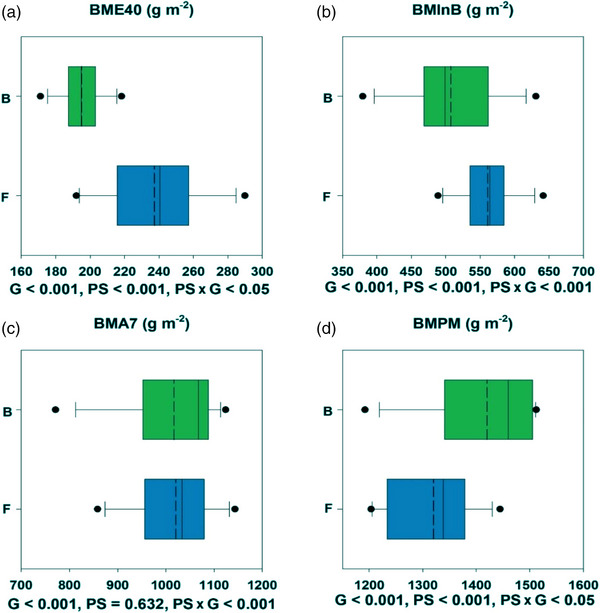
Boxplot of aboveground biomass at (a) emergence + 40 days, (b) initiation of booting, (c) anthesis + 7 days, and (d) physiological maturity in raised beds (B) and flat basins (F). Values represent means across 2017–2018, 2018–2019, and 2019–2020. The middle dotted line is the adjusted mean across lines. Statistical significances for genotype (G), planting systems (PS), and the interaction among them (PS × G) are presented below each boxplot.

Averaging across the 3 years, biomass evaluated at the different growth stages during the season showed genetic variation (*p* < 0.001) (Figure 3). Biomass at 40 days after emergence ranged among the 12 cultivars (*p* < 0.001) from 171 to 218 g m^−2^ in beds and from 192 to 290 g m^−2^ in flats (Figure 3a); and was overall 21.5% higher in flats than in beds (*p* < 0.001). Biomass at initiation of booting was 10.7% higher in flats than beds (*p* < 0.001) with genetic ranges from 376 to 635 g m^−2^ in beds and from 489 to 642 g m^−2^ in flats (Figure 3b). However, biomass at anthesis + 7 days did not differ between planting systems (*p* = 0.63) with cultivars ranging from 771 to 1124 g m^−2^ in beds and from 858 to 1143 g m^−2^ in flats (*p* < 0.001; Figure 3c). At physiological maturity, biomass was 7.6% higher in beds than flats (*p* < 0.001), ranging from 1204 to 1444 g m^−2^ in beds and from 1192 to 1512 g m^−2^ in flats (*p* < 0.001) among cultivars (Figure 3d).

The biomass at each stage showed a statistically significant PS × G interaction. The cultivar SOKOLL showed the greatest biomass increase in beds compared to flats at physiological maturity where beds overall had 226 g m^−2^ more biomass than flats, and the cultivar NELOKI showed the smallest increase. Cultivar SOKOLL was intermediate in the range for plant height among the cultivars with a planophile canopy architecture, whereas cultivar NELOKI was the shortest among the 12 cultivars with an erectophile canopy architecture (Tables 1 and 4). Cultivars showed different temporal patterns of biomass accumulation in the beds and flats during the crop cycle (Figure S2).

**FIGURE 4:**
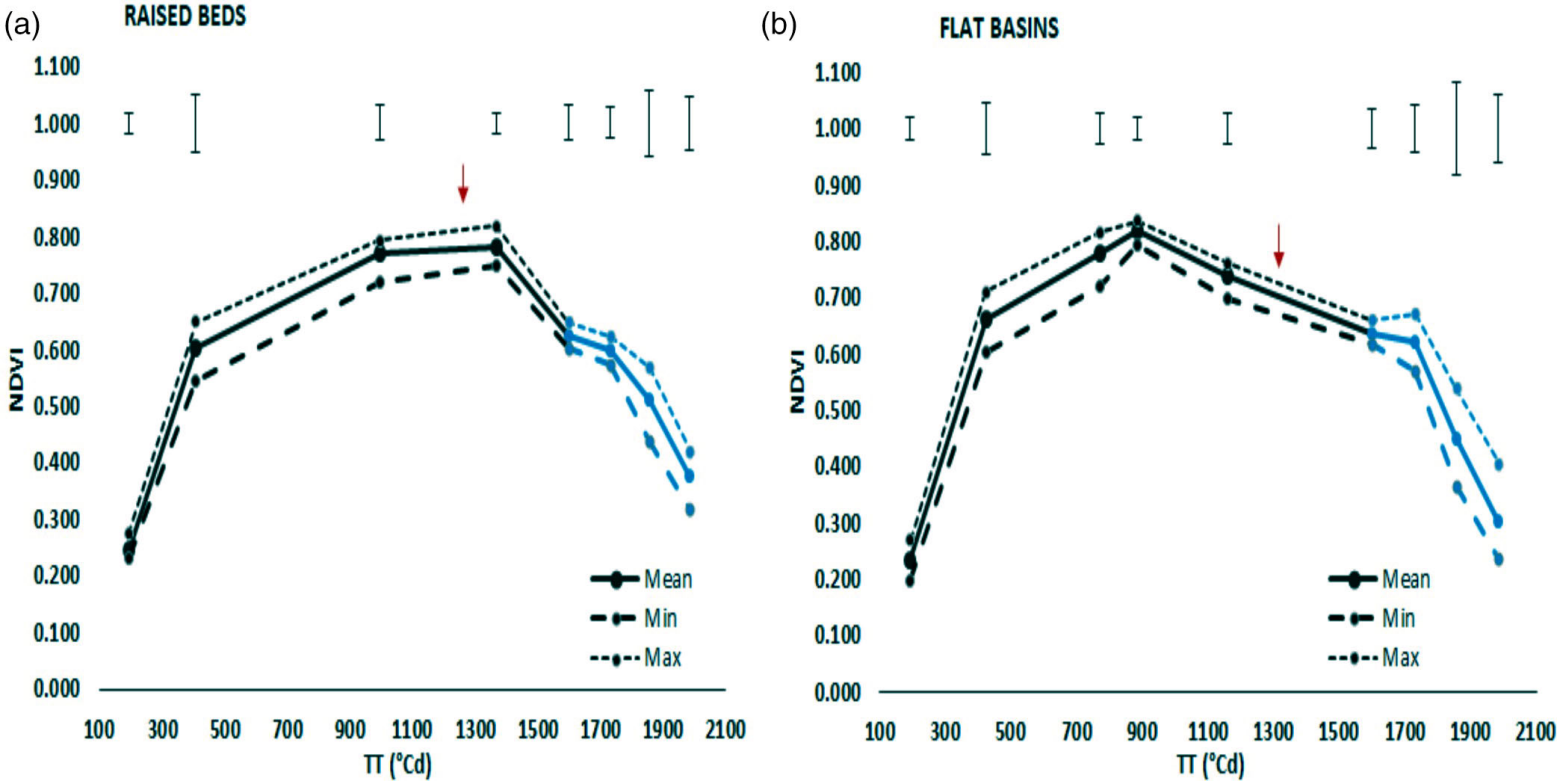
Mean, minimum (Min), and maximum (Max) values of normalized difference vegetation index (NDVI) across 12 CIMMYT spring wheat genotypes with thermal time (TT) from emergence + 40 days on (a) raised beds and (b) flat basins planting systems. Error bars represent the LSD (least significant difference of the means, *p* = 0.05) for each NDVI measurement. The black line represents the vegetative stage cross‐year mean 2017–2018, 2018–2019, and 2019–2020, and the blue line represents the grain‐filing stage cross‐year mean 2017–2018 and 2018–2019. Arrows show the anthesis date.

NDVI during the season in beds and flats is shown in Figure 4. At emergence + 40 days and initiation of booting, both planting systems had similar NDVI values. However, canopy NDVI started to decrease earlier in the flats (at around spike emergence) than in the beds (no decrease from the peak until after anthesis).

**FIGURE 5:**
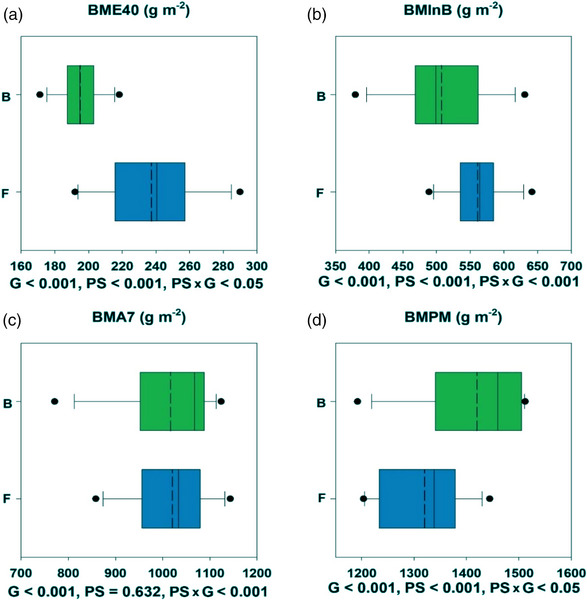
Boxplot of accumulated radiation interception (MJ m^−2^) at (a) emergence + 40 days to the initiation of booting, (b) initiation of booting to anthesis + 7 days, (c) anthesis + 7 days to physiological maturity, and (d) emergence + 40 days to physiological maturity in raised beds (B) and flat basins (F). Values represent means across 2018–2019 and 2019–2020. The middle dotted line is the adjusted mean across lines. Statistical significances for genotype (G), planting systems (PS), and the interaction among them (PS × G) are presented below each boxplot.

Radiation interception and RUE were measured only in 2018–2019 and 2019–2020. Averaging across the 2 years, fractional radiation interception showed genetic variation at GS41 and GS65 + 7 days (*p* < 0.001 and *p* < 0.05, respectively) (Table S4) and was marginally higher in flats than beds at both stages (0.99 vs. 0.98 at GS41 and 0.98 vs. 0.97 at GS65 + 7 days; *p* < 0.001 and *p* < 0.01, respectively). FI at GS65 + 7 days showed a PS × G interaction (*p* < 0.001) with a trend for an interaction at GS41 also (*p* = 0.06). The genetic variation in accumulated intercepted PAR for the phenophases during the crop cycle in both planting systems is shown in Figure 5. In beds, the accumulated intercepted radiation (IPARacc) in the pre‐anthesis phenophases and the grain‐filling phase was slightly greater than in flats. Genetic variation in IPARacc was found for beds and flats for each phenophase (emergence + 40 days–initiation booting, initiation of booting–anthesis + 7 days, anthesis + 7 days–physiological maturity and emergence + 40 days to physiological maturity; *p* < 0.001; Figure 5a–d) and a significant planting system effect (*p* < 0.01, *p* < 0.01, *p* < 0.01, and *p* < 0.001, respectively). The small increases in beds compared to flats for accumulated intercepted radiation were associated with the slightly extended duration of the phenological phases in the beds compared to the flats. A significant PS × G interaction was found for each phenophase (*p* = 0.052, *p* < 0.01, *p* < 0.01, and *p* < 0.05, respectively).

In beds, a strong positive correlation was found between genetic variation in IPARacc from emergence + 40 days to booting and biomass accumulated during this phenophase (*r* = 0.91, *p* < 0.001; Table S5) and similarly for the phenophase from emergence + 40 days to physiological maturity (*r* = 0.72, *p* < 0.01). There was no correlation in flats for these phenophases.

### 3.4 RUE and correlations with biomass and grain yield

For the combined analysis in 2018–2019 and 2019–2020, there were significant effects of planting systems: with RUE early in the season from emergence + 40 days to booting increasing in flats compared to beds (*p* < 0.05) but increases in beds compared to flats from the onset of booting to anthesis + 7 days and over the whole crop cycle from emergence + 40 days to physiological maturity (*p* < 0.05; Table 5). Genetic variation in RUE was statistically significant for each of the phenophases for which RUE was estimated for both beds and flats (*p* < 0.001). For RUE_E40InB, there was a PS × G interaction (*p* < 0.001). The genotypes mainly responsible for the interaction were NELOKI (the highest increase in flats) and ITP40 (the lowest increase in flats). For RUE_InBA7, no PS × G interaction was found. For RUE_preGF, there was a PS × G interaction (*p* < 0.01) for which the cultivars BORLAUG100 and KUKRI were mainly responsible for the highest and lowest increases in beds compared to flats, respectively. There was also an interaction for RUE_GF (*p* < 0.001) with BORLAUG100 and KUKRI having the highest and lowest increases in beds compared to flats, respectively (*p* < 0.001). For RUET, SOKOLL had the greatest increase in beds compared to flats among the cultivars, while NELOKI had the smallest increase (*p* < 0.05).

**TABLE 5:** Radiation‐use efficiency (RUE; g MJ^−1^) calculated from 40 days after emergence to the initiation of booting (RUE_E40InB), from initiation of booting to 7 days after emergence (RUE_InBA7), during the grain‐filling period from 7 days after anthesis to physiological maturity (RUE_GF), pre grain‐filling from 40 days after emergence to 7 days after anthesis (RUE_preGF), and from 40 days after emergence to physiological maturity (RUET) for 12 CIMMYT spring wheat genotypes.

	RUE_E40InB (g MJ^−1^)		RUE_InBA7 (g MJ^−1^)		RUE_GF (g MJ^−1^)		RUE_preGF (g MJ^−1^)		RUET (g MJ^−1^)	
Genotype (G)	B	F	B	F	B	F	B	F	B	F
BACANORA T88	2.03	2.63	3.05	2.76	1.44	1.70	2.20	2.59	1.62	1.61
C80.1/3*QT4118	2.56	2.42	2.78	2.33	1.29	1.17	2.40	2.16	1.71	1.52
CHEWINK#1	2.20	2.44	2.67	2.88	1.93	1.34	2.24	2.46	1.87	1.66
SOKOLL//PUB94	2.40	3.20	3.38	2.47	1.50	1.95	2.58	2.45	1.82	1.85
NELOKI	1.63	2.31	2.64	2.34	1.10	1.19	1.81	2.12	1.30	1.41
W15.92/4/PASTOR	1.99	2.37	3.04	2.32	1.48	1.41	2.23	2.16	1.64	1.56
KUKRI	2.41	2.35	2.47	3.06	1.53	0.67	2.31	2.72	1.78	1.63
KUTZ	2.67	2.77	2.84	2.67	1.32	0.81	2.57	2.45	1.71	1.46
SOKOLL	2.54	2.65	3.51	3.05	1.60	0.72	2.79	2.80	1.98	1.67
BORLAUG100	2.00	2.57	3.59	3.10	1.11	1.51	2.81	2.56	1.91	1.73
ITP40/AKURI	2.52	2.20	3.53	3.17	1.43	1.30	2.64	2.47	1.73	1.61
CHIPAK*2//	2.35	2.42	3.28	3.27	1.13	0.96	2.51	2.76	1.73	1.78
Mean	2.27	2.53	3.06	2.79	1.41	1.23	2.42	2.48	1.73	1.62
LSD (G) (5%)		0.524		0.847		0.740		0.378		0.244
G (*p* value)		^***^		^**^		^**^		^***^		^***^
PS (*p* value)		^*^		^*^		0.145		ns		^*^
Y (*p* value)		0.163		0.115		ns		^*^		ns
PS × G (*p* value)		^***^		0.163		^**^		^**^		^*^

*Note*: Values represent means across 2018–2019 and 2019–2020 in raised beds (B) and flat basins (F) planting systems.

Abbreviations: LSD, least sigfnificant difference of the means, *p* = 0.05; ns, not significant; PS × G, planting system × genotype.

* *p* < 0.05.

** *p* < 0.01.

*** *p* < 0.001.

Genetic variation in grain yield was positively correlated with RUE___preGF in beds (*r* = 0.75, *p* < 0.01) and flats (*r* = 0.70, *p* < 0.05) (Table 6). In addition, yield was positively correlated with RUET in beds (*r* = 0.73, *p* < 0.01) and flats (*r* = 0.67, *p* < 0.05). Grain yield was positively correlated with RUE_InBA7 but only in flats (*r* = 0.87, *p* < 0.001). RUET was positively related with BMPM in beds (*r* = 0.83, *p* < 0.001) and flats (*r* = 0.77, *p* < 0.01). With regard to other traits, in beds, a strong correlation was found between RUE_InBA7 and GM2 (*r* = 0.62, *p* < 0.05) and between RUE_preGF and GM2 (*r* = 0.58, *p* < 0.05).

**TABLE 6:** Phenotypic correlations between yield, yield components, height, aboveground biomass at different growth stages, and shoot number with radiation‐use efficiency (RUE) (g MJ^−1^) for 12 spring CIMMYT wheat genotypes.

	Raised beds (B)					Flat basins (F)				
	RUE_E40InB	RUE_InBA7	RUE_GF	RUE_preGF	RUET	RUE_E40InB	RUE_InBA7	RUE_GF	RUE_preGF	RUET
YLD	0.68^*^	0.37	0.21	0.75^**^	0.73^**^	−0.03	0.87^**^	−0.03	0.70^*^	0.67^*^
GM2	0.01	0.08	−0.08	0.03	−0.02	−0.20	0.62^*^	−0.08	0.58^*^	0.18
HeightPM	0.63^*^	−0.01	0.25	0.41	0.48	0.16	−0.22	−0.13	−0.24	0.06
BMA7	0.72^**^	0.63^*^	−0.07	0.96^***^	0.77^**^	0.01	0.82^***^	−0.61^*^	0.88^***^	0.39
BMPM	0.86^***^	0.37	0.42	0.80^**^	0.83^***^	0.24	0.59^*^	0.05	0.50^****^	0.77^**^

*Note*: Values are based on means from the combined analysis in 2018–2019 and 2019–2020 in raised beds (B) and flat basins (F).

Abbreviations: BMA7, biomass at anthesis + 7 days (g m^−2^); BMPM, biomass at physiological maturity (g m^−2^); GM2, grain number per square meter (m^−2^); HeightPM, plant height at physiological maturity (cm); RUE_E40InB, RUE calculated from 40 days after emergence to the initiation of booting (g MJ^−1^); RUE_InBA7, RUE calculated from initiation of booting to 7 days after emergence (g MJ^−1^); RUE_GF, RUE calculated during the grain‐filling period from 7 days after anthesis to physiological maturity (g MJ^−1^); RUE_preGF, RUE pre grain‐filling from40 days after emergence to 7 days after anthesis (g MJ^−1^); RUET, RUET total, from 40 days after emergence to physiological maturity (g MJ^−1^); YLD, grain yield (g m^−2^).

* *p* < 0.05.

** *p* < 0.01.

*** *p* < 0.001.

**** *p* < 0.10.

### 3.5 Canopy architecture traits and correlations with light interception, RUE, biomass, and grain yield

Stronger correlations between canopy architecture traits and RUE were generally found in flat basins than raised beds. Additionally, stronger correlations were found using the quantitative methodology to measure flag‐leaf curvature rather than the qualitative visual score (Table 7). The correlations between flag‐leaf angle and RUE contrasted in the two planting systems. In flat basins, there was a negative correlation with more upright leaves at GS65 + 7 days correlated with higher RUE_GF (*p* < 0.05), whereas in raised beds, there were positive correlations between flag‐leaf angle at the onset of booting and RUE_preGF (*p* < 0.10) and RUET (*p* < 0.05) (Table 7). For leaf curvature measurements, there was a positive correlation between the visual score of flag‐leaf curvature in beds at each of GS41 (*r* = 0.65, *p* < 0.05) and GS65 + 7 days (*r* = 0.59, *p* < 0.05) and RUET. There was also a positive correlation in beds between the quantitative measure of flag‐leaf curvature at GS65 + 7 days and RUE_GF (*r* = 0.78, *p* < 0.0). A positive correlation was also found between flag‐leaf relative chlorophyll content in leaf 3 at GS65 + 7 days and RUE_preGF in raised beds (*R* ^2^ = 0.54, *p* < 0.01) and flat basins (*R* ^2^ = 0.43, *p* < 0.05) (Table 7).

**TABLE 7:** Phenotypic correlations among 12 CIMMYT spring wheat genotypes evaluated across the years 2018–2019 and 2019–2020 in raised beds and flat basins between canopy architecture and physiological traits using different methodologies (quantitative flag‐leaf angle and curvature/qualitative visual score).

	Raised beds (B)						Flat basins (F)					
	Quantitative methods				Qualitative visual score		Quantitative methods				Qualitative visual score	
	FLAInB	FLCInB	FLAA7	FLCA7	FLvscInB	FLvscA7	FLAInB	FLCInB	FLAA7	FLCA7	FLvscInB	FLvscA7
YLD	0.45	0.07	0.36	−0.10	0.22	0.27	−0.16	0.41	−0.07	−0.19	0.12	−0.03
TGW	−0.75^**^	−0.55^****^	0.53^****^	−0.37	0.74^**^	0.73^*^	0.65^*^	−0.56^****^	0.46	−0.20	0.70^*^	0.67^*^
GM2	−0.48	0.65^*^	−0.33	0.38	−0.63^*^	−0.61^*^	−0.64^*^	0.66^*^	−0.46	−0.20	−0.54^****^	−0.60^*^
BMPM	0.60^*^	−0.27	0.36	−0.34	0.53^****^	0.54^****^	0.30	0.14	0.10	−0.21	0.34	0.28
RUE_InBA7	−0.05	0.23	−0.13	0.23	−0.04	−0.08	−0.38	0.62^*^	−0.01	−0.31	−0.03	−0.09
RUE_GF	0.19	−0.17	−0.24	0.08	0.30	0.36	0.08	−0.25	−0.68^*^	0.78^**^	−0.11	−0.13
RUE_preGF	0.51^****^	−0.15	0.38	−0.27	0.42	0.34	−0.21	0.40	0.06	−0.49	0.12	−0.00
RUET	0.64^*^	−0.27	0.35	−0.16	0.65^*^	0.59^*^	0.08	0.15	−0.18	−0.02	0.34	0.25
FLIInB	0.67^*^	−0.45	0.45	−0.39	0.52^****^	0.34	0.35	−0.23	0.43	−0.53^****^	0.63^*^	0.57^****^
FLIA7	0.58^*^	−0.25	0.34	−0.17	0.52^****^	0.48	−0.08	−0.11	−0.25	0.28	−0.17	−0.20
HeightPM	0.70^**^	−0.64^*^	0.59^*^	−0.52^****^	0.71^**^	0.67^*^	0.74^**^	−0.44	0.67^*^	−0.34	0.56^****^	0.60^*^
SPAD3	0.35	−0.14	0.12	−0.03	0.32	0.25	0.02	0.27	0.02	−0.30	0.07	−0.08

Abbreviations: BMPM, biomass at physiological maturity (g m^−2^); FLAA7: flag‐leaf angle at 7 days after anthesis (°); FLAInB, flag‐leaf angle at initiation of booting (°); FLIA7, light interception at anthesis + 7 days; FLIInB, light interception at initiation of booting; FLcvInB, the distance from the point of inflexion of the flag‐leaf to the tip at the initiation of booting (cm); FLcvA7, the distance from the point of inflecion of the flag‐leaf to the tip at 7 days after anthesis (cm); FLvscInB, flag‐leaf curvature score at the initiation of booting; FLvscA7, flag‐leaf curvature score at anthesis + 7 days; GM2, grain number per square meter (m^−2^); HeightPM, plant height at physiological maturity (cm); RUE_GF, RUE calculated during the grain‐filling period from 7 days after anthesis to physiological maturity (g MJ^−1^); RUE_InBA7, RUE calculated from initiation of booting to 7 days after emergence (g MJ^−1^); RUE_preGF, RUE pre grain‐filling from40 days after emergence to 7 days after anthesis (g MJ^−1^); RUET, RUET total, from 40 days after emergence to physiological maturity (g MJ^−1^); SPAD3, SPAD in leaf 3; TGW, thousand‐grain weight; YLD, grain yield (g m^−2^).

* *p* < 0.05.

** *p* < 0.01.

*** *p* < 0.001.

**** *p* < 0.10.

With regard to light interception, in raised beds, a positive correlation was found between flag‐leaf angle at the initiation of booting and fractional light interception at each of GS41 (*r* = 0.67, *p* < 0.05) and GS65 + 7 days (*r* = 0.58. *p* < 0.05). There were no correlations between flag‐leaf angle and fractional light interception in flat basins. A correlation was found between the quantitative flag‐leaf curvature measurements at GS41 and FI at GS41 (*r* = 0.67, *p* < 0.05) and at G65 + 7 days (*r* = 0.58, *p* < 0.05) in beds. Similarly, a positive correlation was found between the visual score of curvature at the initiation of booting and FI at GS65 + 7 days in raised beds (0.52, *p* = 0.08) and flat basins (*r* = 0.63, *p* < 0.05). Fractional light interception at anthesis and booting was strongly correlated with biomass at anthesis + 7 in raised beds (r > 0.70; *p* < 0.01), but there were no correlations in flat basins (Table S6).

The phenotypic correlations between canopy architecture traits and grain yield, biomass, and yield components in raised beds and flat basins are shown in Table 7. A positive association between flag‐leaf angle at GS41 and biomass at physiological maturity was found in raised beds (*r* = 0.60, *p* < 0.05); there was no correlation in raised beds.

## 4 DISCUSSION

### 4.1 Effects on planting system and cultivar

The irrigated wheat production in North West Mexico has adopted a raised‐bed planting system over the last decades which has increased the efficiency of the use of irrigation water and fertilizer N and provided other management benefits over flat basins (Fahong et al., 2004; Fischer, 2005). Present results of higher grain yield in beds than flats (10.6%) were similar to several previous investigations reporting yield gains in beds compared to flat basins (Fahong et al., 2004; Hassan et al., 2005; Kakar et al., 2015; Majeed et al., 2015; Ram et al., 2005). Nevertheless, Tanveer et al. (2003) and López‐Castañeda et al. (2014) found greater grain yield in flats than beds.

We identified several physiological reasons for the higher grain yield in beds than flats in our experiments. RUE from the initiation of booting to 7 days after anthesis was greater in beds by 9.7% as well as season‐long RUE by 6.8% contributing to greater aboveground biomass (7.6%) at physiological maturity in beds than flats. Higher RUE from booting to anthesis + 7 days allowed the beds to catch up with the flats in biomass at anthesis + 7 days. The higher RUE from booting to anthesis + 7 days may have been partly associated with an effect for more upright flag‐leaf angle in beds at 6° compared to flats at 13° at booting reducing light saturation of photosynthesis in the flag leaves. On the other hand, at GS65 + 7 days, there was a small increase for flag‐leaf angle in beds compared to flats (72° for beds vs. 68° for flats). The higher RUE in beds than flats may also have been associated with the wider row spacing in raised beds compared to flat basins (24 vs. 20 cm) allowing more light to penetrate to lower leaf layers and reducing light saturation of the flag leaf. The higher grain yield in beds was also, in part, related to an extended grain‐filling duration by 2 days in beds compared with flats, which contributed to higher post‐anthesis radiation interception in beds. It can be speculated that beds led to slightly cooler canopies hence delayed phenology, although canopy temperature was not measured in the present study. Higher post‐anthesis radiation interception and biomass accumulation in beds than flats were additionally associated with a delayed onset of NDVI senescence (Figure 4) in beds compared to flats. There was no apparent evidence that the higher biomass in beds than flats was associated with hypoxia in the flat plots; in all years, there were no visible symptoms of hypoxia in any of the plots in either the raised beds or flat basins throughout the life cycle. There was no planting system effect on flag‐leaf chlorophyll content (data not shown), in contrast with a previous study where leaf SPAD was higher in raised beds than flat basins associated with higher N uptake (Fahong et al., 2004).

### 4.2 Genetic variation in radiation interception and RUE in planting systems and associations with canopy architecture traits

RUE showed genetic variation in the different phenophases during the season with broadly similar ranges to previous field studies. For example, a study in winter wheat in the United Kingdom found genetic variation of RUE based on PAR in the range 2.33–2.63 g MJ^−1^ under rain‐fed conditions (Shearman et al., 2005), and a study in bread wheat in Spain reported values based on solar radiation from 0.85 to 1.54 g MJ^−1^ from anthesis to maturity under irrigated conditions (Acreche et al., 2009). Genetic variation in RUE_preGF showed an association with biomass at GS65 + 7 days and physiological maturity in both planting systems. In addition, our results showed that genetic variation in RUE_preGF was positively correlated with grain yield in beds and flats. Previous studies by Tao et al. (2018) and Molero et al. (2019) in wheat also demonstrated correlations between pre‐anthesis RUE and grain yield among genotypes. In our study, the correlation between RUE and biomass was stronger in flats than beds.

There was a strong negative association between genetic variation in the flag‐leaf angle at GS65 + 7 days and RUE_GF in flat basins with more upright leaves leading to higher RUE, which agrees with Reynolds et al. (2000) and Murchie et al. (2009). Previous work has shown that irradiance incident on the top of the canopy saturates the flag‐leaf less in erectophiles compared to planophiles canopies in wheat (Araus et al., 1993; Hirose, 2005) and rice (Chang et al., 2019). In addition, there was a positive association between flag‐leaf curvature at GS65 + 7 days and RUE_GF in flat basins which could imply that more recurved flag‐leaves allowed more light to reach the lower leaf layers and hence increased RUE. However, no correlation was found between the fraction of light intercepted at the bottom of the canopy and flag‐leaf curvature for either planting system (data not shown). In flat basins, no correlations were found between flag‐leaf angle and fractional light interception.

When effects in raised beds were considered, in the present study, there was no correlation between flag‐leaf angle and RUE in raised beds. It is likely that more upright leaves were relatively more advantageous for RUE in flats than beds due to the narrower row spacing in flats predisposing the flag leaves more to light saturation in upper leaf layers than in beds. There was, however, a positive correlation between flag‐leaf angle and fractional light interception at GS41 in raised beds (cultivars with less upright leaves had higher fractional light interception). Cultivars with less upright flag‐leaf angle at GS41 also had greater biomass at physiological maturity in raised beds likely related to the increased light interception pre‐anthesis. Therefore, results indicated that cultivars with less upright flag leaves were better at capturing light in raised beds translating to a greater biomass at harvest. Again, this planting system difference could be partly explained by row spacing effects, with the greater row spacing (and the gap between the beds in the raised beds) potentially favoring lax‐leaved genotypes in this planting system since they can intercept more light per unit leaf area (Fischer et al., 2019). No association between narrower flag‐leaves and increased SPAD at 7 days after anthesis was found for either planting system (data not shown). Interestingly, in both planting systems, chlorophyll content in leaf 3 (flag leaf = leaf 1) was positively associated among cultivars with RUE_preGF (Table 7). The vertical distribution of N in leaf layers may not be optimized in modern cultivars with insufficient N in lower layers to maximize RUE (Foulkes & Murchie, 2011). In addition, higher chlorophyll concentration may relate to more light‐harvesting complexes to improve light capture in lower leaves (Townsend et al., 2018). Further work is required on the relation between leaf N traits and RUE to understand the critical value of leaf N in the respective leaf layers at which RUE is maximized in raised beds and flat basins.

### 4.3 Cultivar responses to planting systems and implications for plant breeders

A significant PS × G interaction was found for grain yield; for example, the genotype C80.1/3*QT4118 had 18.2% higher grain yield in beds than flats, whereas the genotype ITP40 showed the same grain yield in both planting systems. The grain yield responses to the planting system were mainly driven by the aboveground biomass responses at physiological maturity rather than HI. Regarding yield components, grain yield responses to PS were determined by grains m^−2^ responses rather than TGW responses. Taller cultivars intercepted relatively more radiation in the beds than the flats before anthesis, consistent with the taller cultivars showing relatively greater increases in grain yield and biomass at physiological maturity in beds compared to flats. Aboveground biomass at each stage showed a PS × G interaction. However, differences in radiation interception were not the only ones explaining differences in genotype responses to PS for biomass.

The cultivar responses to planting system for biomass at 7 days after anthesis were associated with responses for RUE_InBA7 but more strongly with responses for RUE_preGF. Additionally, responses of cultivars to PS for biomass at physiological maturity were associated with responses to PS for RUET. In summary, the present results in flat basins indicate that breeders should focus on leaf angle to avoid light saturation of leaves, hence increase RUE and grain yield in plant breeding programs where this trait is not already optimized. In raised beds, however, the case for more upright leaves was not clearly demonstrated: on the contrary, cultivars with less upright leaves had higher fractional light interception in the pre‐anthesis phase and higher biomass at maturity. A study in sorghum proposed a “smart canopy” with upright leaves at the top combined with horizontal leaves at the bottom of the canopy to maximize light interception (Mantilla‐Perez et al., 2020), and it is feasible that such a canopy type would be beneficial in raised beds.

Plant breeders have selected wheat canopies with smaller flag leaves and more upright leaves in recent years in some countries, for example, in winter wheat in the United Kingdom (Shearman et al., 2005). The present results indicated that the genotypes with upright leaves allowed a higher RUE during grain filling and biomass in flat basins, which is the most common wheat planting system globally. Additionally, the results suggested that more prostrate leaves in raised beds may be beneficial for increasing biomass through increased light interception. Furthermore, the results showed that it is important to measure canopy architecture traits at more than one phenological stage, since the effect of planting systems differed for some traits between development stages, for example, for flag‐leaf angle between booting and anthesis + 7 days.

Future work will require more genetic studies to better understand the genes regulating the canopy architecture traits. The present results indicated that the future selection of cultivars with less upright but more recurved leaves may be more beneficial for raising light interception and RUE and biomass in raised beds, but selection for more upright leaves would be more beneficial in flat basins. There is a need for further studies on how the canopy architecture traits affect RUE in different planting systems and mega‐environments in a wider range of germplasm in order to reinforce and augment the present findings.

## 5 CONCLUSIONS

Planting systems play an important role in determining biomass, yield, and RUE in wheat. The results indicated that the raised beds were beneficial for wheat production in North West Mexico compared to the flat basins justifying its wide use by farmers in the region. In addition, planophile types were associated with higher fractional light interception in beds, while erect types were associated with higher RUE in flats‐basin. This explains why most of the spring bread wheat from CIMMYT have planophile architecture as the bed system has been predominantly used in the bread wheat breeding program. Yield was highly correlated with RUE_preGF in both planting systems suggesting that this can be a target trait to select for both planting systems. Based on the present results, the evaluation of genotypes for RUE, as well as other traits such as biomass, should take into account the different planting systems, and evaluations in the system of the target environment will be required. However, if selections are targeted for multiple planting systems, it will be necessary to capture genotype × planting system effects and select genotypes that perform better in both systems (e.g., ITP40 in this study).

## AUTHOR CONTRIBUTIONS

**Marcela A. Moroyoqui‐Parra**: Formal analysis; investigation; methodology; writing—original draft; writing—review and editing. **Gemma Molero**: Investigation; methodology; writing—review and editing. **Matthew P. Reynolds**: Conceptualization; funding acquisition; writing—review and editing. **Oorbessy Gaju**: Methodology; writing—review and editing. **Erik H. Murchie**: Methodology; writing—review and editing. **Michael John Foulkes**: Conceptualization; funding acquisition; methodology; writing—original draft; writing—review and editing.

## CONFLICT OF INTEREST STATEMENT

The authors declare no conflicts of interest.

## Supporting information


**Supplementary Table 1**. Growing conditions for three seasons (2016‐17, 2017–18 and 2018–19) for field experiments in the planting systems (PS), raised beds (B) and flat basins (F).
**Supplementary Table 2**. ANOVA for grain yield (YLD), 1,000 grain weight (TGW), harvest index (HI), grains m^−2^ (GM2) and above‐ground biomass at physiological maturity (BMPM), plant height at physiological maturity (HeightPM) and date of anthesis (GS65, DTA) from the combined analysis across 2017–18, 2018–19 and 2019–20 in raised beds (B) and flat basins (F).
**Supplementary Table 3**. Flag‐leaf length and width at initiation of booting (GS41) and seven days after anthesis (GS65) and SPAD in the leaf 3 at seven days after anthesis for 12 CIMMYT spring wheat cultivars from the combined analysis across 2018–19 and 2019–20 in raised beds (B) and flat basins (F).
**Supplementary Table 4**. Mean, minimum, maximum, and ANOVA for fractional light interception at emergence + 40 days (FLI.E40), initiation of booting (FLI.InB) and seven days after anthesis (FLI.A7) from the combined analysis across 2018–19 and 2019–20 in raised beds (B) and flat basins (F). *p < 0.05, **p < 0.01, ***p < 0.001, *italics: P* < *0.10*, ns: not significant.
**Supplementary Table 5**. Phenotypic correlations between IPARacc for each phenophase and above‐ground biomass at different growth stages and plant height at physiological maturity for 12 spring CIMMYT wheat genotypes. Values based on means from the combined analysis in 2018–19 and 2019–20 in raised beds (B) and flat basins (F). *P < 0.05, **P < 0.01, ***P < 0.001, †P < 0.10.
**Supplementary Table 6**. Phenotypic correlations between fractional light interception (FLI) and above‐ground biomass at different growth stages for 12 spring CIMMYT wheat genotypes. Values based on means from the combined analysis in 2018–19 and 2019–20 in raised beds (B) and flat basins (F).
**Supplementary Table 7**. Broad‐sense heritability (H^2^) for yield, yield components, biomass at maturity, phenology expressed in days after emergence (DAE), number of shoots from the three combined analysis across 2017–18, 2018–19 and 2019–20 and RUE from the two combined analysis (2018‐19 and 2019–20). Plants (m^−2^)‡ (data 2019–20).
**Supplementary Figure 1**. Environmental conditions in the field experiments (average daily mean temperature (°C), average daily minimum temperature (Tmin, °C), average daily maximum temperature (Tmax, °C), monthly rainfall (mm) and average daily radiation (MJ m^−2^) in the field experiments during (A) 2017–18, (B) 2018–19 and (C) 2019–20.
**Supplementary Figure 2**. Above‐ground biomass accumulation during the crop cycle for 12 CIMMYT spring wheat genotypes evaluated across‐years in 2017–18, 2018–19 and 2019–20 in raised beds (B) and flat basins (F). TT = thermal time post‐emergence. *P < 0.05.
