# Supplementary material for: Interaction of planting system with radiation‐use efficiency in wheat lines
Source: Crop Sci. 2023 Nov 14;64(1):314–32. doi: 10.1002/csc2.21115 (PMC10952436; doi:10.1002/csc2.21115)
Supplement: Supplementary file 1 — Supplementary Table 1. Growing conditions for three seasons (2016‐17, 2017–18 and 2018–19) for field experiments in the planting systems (PS), raised beds (B) and flat basins (F). Supplementary Table 2. ANOVA for grain yield (YLD), 1,000 grain weight (TGW), harvest index (HI), grains m−2 (GM2) and above‐ground biomass at physiological maturity (BMPM), plant height at physiological maturity (HeightPM) and date of anthesis (GS65, DTA) from the combined analysis across 2017–18, 2018–19 and 2019–20 in raised beds (B) and flat basins (F). Supplementary Table 3. Flag‐leaf length and width at initiation of booting (GS41) and seven days after anthesis (GS65) and SPAD in the leaf 3 at seven days after anthesis for 12 CIMMYT spring wheat cultivars from the combined analysis across 2018–19 and 2019–20 in raised beds (B) and flat basins (F). Supplementary Table 4. Mean, minimum, maximum, and ANOVA for fractional light interception at emergence + 40 days (FLI.E40), initiation of booting (FLI.InB) and seven days after anthesis (FLI.A7) from the combined analysis across 2018–19 and 2019–20 in raised beds (B) and flat basins (F). *p < 0.05, **p < 0.01, ***p < 0.001, italics: P < 0.10, ns: not significant. Supplementary Table 5. Phenotypic correlations between IPARacc for each phenophase and above‐ground biomass at different growth stages and plant height at physiological maturity for 12 spring CIMMYT wheat genotypes. Values based on means from the combined analysis in 2018–19 and 2019–20 in raised beds (B) and flat basins (F). *P < 0.05, **P < 0.01, ***P < 0.001, †P < 0.10. Supplementary Table 6. Phenotypic correlations between fractional light interception (FLI) and above‐ground biomass at different growth stages for 12 spring CIMMYT wheat genotypes. Values based on means from the combined analysis in 2018–19 and 2019–20 in raised beds (B) and flat basins (F). Supplementary Table 7. Broad‐sense heritability (H2) for yield, yield components, biomass at maturity, phenology expressed [file CSC2-64-314-s001.docx]

**Supplemental information**

**Supplementary Table 1**. Growing conditions for three seasons (2016-17, 2017-18 and 2018-19) for field experiments in the planting systems (PS), raised beds (B) and flat basins (F).

| **Crop Cycle** | **PS** | **Sowing**  **date** | **N application** | **P application** | **Irrigations (no.)** |
| --- | --- | --- | --- | --- | --- |
|  |  |  | kg ha^-1^ | kg ha^-1^ |  |
| 2017-2018 | B | 30/11/2017 | 50-200-50 | 50 | 4 |
|  | F | 01/12/2017 | 50-200-50 | 50 | 5 |
| 2018-2019 | B | 30/11/2018 | 50-200 | 50 | 6 |
|  | F | 01/12/2018 | 50-200 | 50 | 6 |
| 2019-2020 | B | 21/12/2019 | 50-200 | 50 | 6 |
|  | F | 17/12/2019 | 50-200 | 50 | 9 |


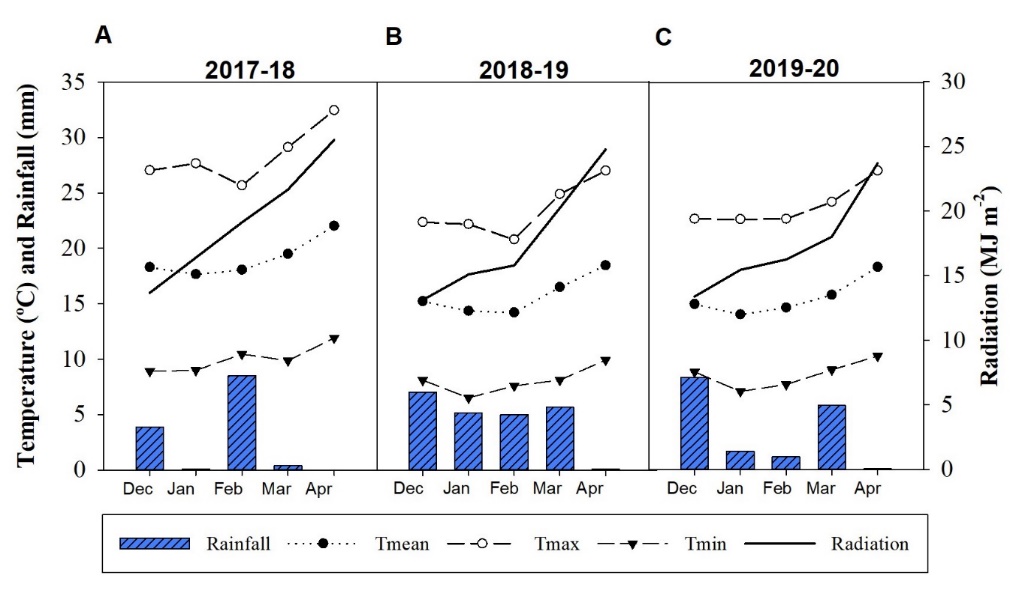


**Supplementary Figure 1.** Environmental conditions in the field experiments (average daily mean temperature (°C), average daily minimum temperature (Tmin, °C), average daily maximum temperature (Tmax, °C), monthly rainfall (mm) and average daily radiation (MJ m^-2^) in the field experiments during (A) 2017-18, (B) 2018-19 and (C) 2019-20.

**Supplementary Table 2.** ANOVA for grain yield (YLD), 1,000 grain weight (TGW), harvest index (HI), grains m^-2^ (GM2) and above-ground biomass at physiological maturity (BMPM), plant height at physiological maturity (HeightPM) and date of anthesis (GS65, DTA) from the combined analysis across 2017-18, 2018-19 and 2019-20 in raised beds (B) and flat basins (F).

| Genotype (G) | YLD | | TGW | | HI | | GM2 | | BMPM | | HeightPM | | DTA | |
| --- | --- | --- | --- | --- | --- | --- | --- | --- | --- | --- | --- | --- | --- | --- |
|  | g m^-2^ | | g | |  | | m^-2^ | | g m^-2^ | | cm | |  | |
|  | B | F | B | F | B | F | B | F | B | F | B | F | B | F |
| BACANORA T88 | 634 | 591 | 35.18 | 35.20 | 0.50 | 0.49 | 18067 | 16785 | 1281 | 1204 | 90.1 | 88.3 | 75 | 75 |
| C80.1/3*QT4118 | 668 | 565 | 47.85 | 46.82 | 0.44 | 0.42 | 13958 | 12092 | 1494 | 1338 | 121.3 | 119.4 | 81 | 80 |
| CHEWINK#1 | 683 | 610 | 46.61 | 45.70 | 0.47 | 0.44 | 14654 | 13377 | 1459 | 1396 | 109.0 | 110.0 | 79 | 78 |
| SOKOLL//PUB94 | 680 | 592 | 51.21 | 50.44 | 0.44 | 0.43 | 13284 | 11730 | 1512 | 1373 | 111.3 | 112.8 | 77 | 76 |
| NELOKI | 539 | 525 | 36.64 | 37.32 | 0.45 | 0.44 | 14800 | 14109 | 1192 | 1210 | 93.7 | 95.4 | 74 | 74 |
| W15.92/4/PASTOR | 593 | 547 | 51.20 | 51.57 | 0.45 | 0.45 | 11584 | 10611 | 1324 | 1219 | 107.9 | 108.1 | 72 | 72 |
| KUKRI | 695 | 607 | 44.07 | 44.63 | 0.49 | 0.47 | 15792 | 13637 | 1407 | 1290 | 106.1 | 106.8 | 78 | 76 |
| KUTZ | 696 | 605 | 47.54 | 46.66 | 0.46 | 0.45 | 14654 | 12995 | 1508 | 1338 | 111.1 | 110.7 | 79 | 78 |
| SOKOLL | 638 | 561 | 44.91 | 44.30 | 0.43 | 0.44 | 14251 | 12662 | 1503 | 1277 | 108.2 | 106.2 | 76 | 75 |
| BORLAUG100 | 730 | 682 | 47.78 | 47.71 | 0.50 | 0.50 | 15300 | 14301 | 1460 | 1381 | 102.3 | 102.5 | 75 | 75 |
| ITP40/AKURI | 741 | 646 | 46.16 | 45.63 | 0.49 | 0.48 | 16029 | 14158 | 1505 | 1371 | 104.6 | 107.5 | 78 | 77 |
| CHIPAK*2// | 693 | 693 | 42.89 | 41.99 | 0.50 | 0.48 | 16227 | 16511 | 1393 | 1444 | 102.6 | 100.3 | 78 | 77 |
| Mean | 666 | 602 | 45.17 | 44.83 | 0.47 | 0.46 | 14883 | 13581 | 1420 | 1320 | 105.7 | 105.7 | 77 | 76 |
| H^2^ | 0.90 | | 0.95 | | 0.94 | | 0.97 | | 0.86 | | 0.82 | | 0.45 | |
| LSD (G) (5%) | 68.239 | | 2.227 | | 0.035 | | 1659.615 | | 165.653 | | 3.916 | | 1.622 | |
| CV% | 6.79 | | 3.05 | | 4.73 | | 7.37 | | 7.55 | | 2.28 | | 1.32 | |
| G (p-value) | ******* | | ******* | | ******* | | ******* | | ******* | | ******* | | ******* | |
| PS (p-value) | ******* | | *0.097* | | ***** | | ******* | | ******* | | ns | | ******* | |
| Y (p-value) | ***** | | ******* | | ******* | | ***** | | ****** | | ******* | | ******* | |
| PS×G (p-value) | ***** | | ns | | ns | | 0.184 | | ***** | | ***** | | *0.084* | |


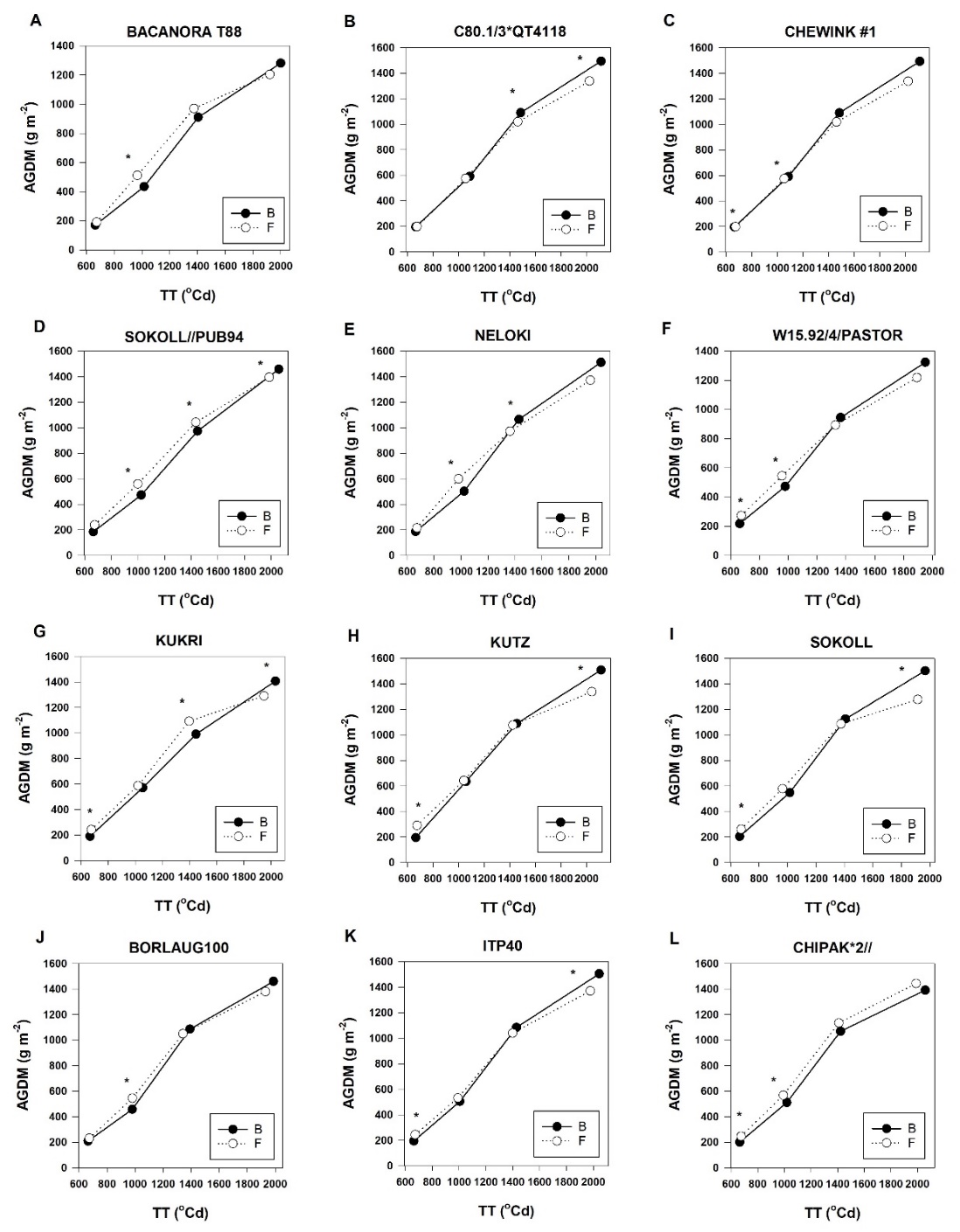


**Supplementary Figure 2.** Above-ground biomass accumulation during the crop cycle for 12 CIMMYT spring wheat genotypes evaluated across-years in 2017-18, 2018-19 and 2019-20 in raised beds (B) and flat basins (F). TT = thermal time post-emergence. *P < 0.05.

**Supplementary Table 3.** Flag-leaf length and width at initiation of booting (GS41) and seven days after anthesis (GS65) and SPAD in the leaf 3 at seven days after anthesis for 12 CIMMYT spring wheat cultivars from the combined analysis across 2018-19 and 2019-20 in raised beds (B) and flat basins (F).

|  | Initiation of booting | | | | Anthesis + 7 days | | | | | |
| --- | --- | --- | --- | --- | --- | --- | --- | --- | --- | --- |
| Genotype | LLInB  (cm) | | LWInB  (cm) | | LLA7  (cm) | | LWA7  (cm) | | SPAD  Leaf3 | |
|  | B | F | B | F | B | F | B | F | B | F |
| BACANORA T88 | 24.9 | 22.0 | 1.8 | 1.8 | 25.9 | 22.0 | 1.7 | 1.7 | 43.43 | 45.20 |
| C80.1/3*QT4118 | 32.5 | 34.8 | 2.1 | 2.0 | 34.6 | 31.9 | 2.0 | 2.0 | 44.88 | 44.73 |
| CHEWINK#1 | 35.8 | 28.4 | 2.2 | 1.9 | 34.8 | 29.0 | 2.0 | 2.0 | 44.93 | 46.88 |
| SOKOLL//PUB94 | 28.9 | 24.3 | 1.9 | 1.9 | 31.2 | 25.2 | 1.8 | 1.8 | 49.10 | 46.15 |
| NELOKI | 24.9 | 24.2 | 1.8 | 2.0 | 28.7 | 23.0 | 1.8 | 1.9 | 42.42 | 43.73 |
| W15.92/4/PASTOR | 27.2 | 23.7 | 2.0 | 2.0 | 27.9 | 22.6 | 1.9 | 1.9 | 43.80 | 44.12 |
| KUKRI | 27.8 | 25.5 | 1.9 | 1.8 | 28.9 | 23.1 | 1.8 | 1.7 | 44.02 | 45.70 |
| KUTZ | 26.8 | 24.9 | 1.9 | 1.9 | 27.3 | 25.0 | 1.9 | 2.0 | 44.97 | 47.33 |
| SOKOLL | 28.6 | 24.4 | 1.8 | 1.8 | 30.3 | 24.6 | 1.7 | 1.8 | 47.80 | 46.53 |
| BOURLAG100 | 32.9 | 26.5 | 2.1 | 2.0 | 32.3 | 27.6 | 1.9 | 2.0 | 46.35 | 47.15 |
| ITP40/AKURI | 23.9 | 21.6 | 1.8 | 1.8 | 24.8 | 22.0 | 1.7 | 1.8 | 45.95 | 48.27 |
| CHIPAK*2// | 25.7 | 23.0 | 1.9 | 1.9 | 26.3 | 21.2 | 1.9 | 1.8 | 47.50 | 49.62 |
| Mean | 23.9 | 25.3 | 1.9 | 1.9 | 29.4 | 24.8 | 1.8 | 1.9 | 45.43 | 49.62 |
| H^2^ | 0.79 | | 0.78 | | 0.65 | | 0.68 | | 0.77 | |
| LSD (G) (5%) | 3.69 | | 0.15 | | 3.75 | | 0.16 | | 3.24 | |
| CV% | 8.48 | | 4.79 | | 8.53 | | 5.41 | | 4.35 | |
| G (p value) | ******* | | ******* | | ******* | | ******* | | ******* | |
| PS (p value) | ******* | | 0.128 | | ******* | | 0.150 | | ***** | |
| Y (p value) | ***** | | ******* | | ***** | | ns | | ns | |
| PS×G (p value) | ******* | | ******* | | ns | | *0.065* | | ***** | |

LLInB: flag-leaf length at initiation of booting (cm), LW: flag-leaf width at initiation of booting (cm), LLA7: flag-leaf length at seven days after anthesis (cm), LWA7: flag-leaf width at seven days after anthesis (cm), SPAD.leaf3: SPAD in the leaf 3 *P < 0.05, **P < 0.01, ***P < 0.001, *italics*: *P < 0.10*, ns: not significant.

|  |  | |  | |  | | p-value | | | |
| --- | --- | --- | --- | --- | --- | --- | --- | --- | --- | --- |
| Trait | Mean | | Min | | Max | | G | Y | PS | PS×G |
|  | B | F | B | F | B | F |  |  |  |  |
| FLI.E40 | 0.88 | 0.90 | 0.87 | 0.89 | 0.90 | 0.93 | ns | ****** | 0.122 | ns |
| FLI.InB | 0.98 | 0.99 | 0.96 | 0.98 | 0.98 | 0.99 | ******* | ******* | ******* | *0.064* |
| FLI.A7 | 0.97 | 0.98 | 0.94 | 0.97 | 0.98 | 0.99 | ***** | ****** | ****** | ******* |

**Supplementary Table 4.** Mean, minimum, maximum, and ANOVA for fractional light interception at emergence + 40 days (FLI.E40), initiation of booting (FLI.InB) and seven days after anthesis (FLI.A7) from the combined analysis across 2018-19 and 2019-20 in raised beds (B) and flat basins (F). *P < 0.05, **P < 0.01, ***P < 0.001, *italics: P < 0.10,* ns: not significant.

|  | RAISED BEDS (B) | | | | FLAT BASINS (F) | | | |
| --- | --- | --- | --- | --- | --- | --- | --- | --- |
|  | IPARacc_E40-InB | IPARacc_  InB-A7 | IPARacc__A7-PM | IPARacc__E40-PM | IPARacc_E40-InB | IPARacc_  InB-A7 | IPARacc__A7-PM | IPARAacc__E40-PM |
| HeightPM | **0.68*** | -0.11 | 0.34 | 0.51† | 0.50**†** | 0.34 | -0.04 | 0.35 |
| BMaccE40_InB | **0.91***** | -0.24 | 0.55† | **0.72**** | 0.44 | 0.20 | 0.12 | 0.38 |
| BMaccInB_A7 | -0.44 | 0.46 | -0.34 | -0.33 | 0.28 | **0.64*** | -0.36 | 0.16 |
| BMaccA7_PM | 0.25 | 0.28 | 0.12 | 0.25 | -0.24 | -0.29 | 0.17 | -0.12 |

**Supplementary Table 5.** Phenotypic correlations between IPARacc for each phenophase and above-ground biomass at different growth stages and plant height at physiological maturity for 12 spring CIMMYT wheat genotypes. Values based on means from the combined analysis in 2018-19 and 2019-20 in raised beds (B) and flat basins (F). *P < 0.05, **P < 0.01, ***P < 0.001, †P < 0.10.

HeightPM: plant height at physiological maturity (cm), BMaccE40_InB: biomass accumulated from emergence + 40 days to initiation of booting (g m^-2^), BMaccInB_A7: biomass accumulated from initiation of booting to anthesis + 7 days (g m^-2^), BMaccA7_PM: biomass accumulated from anthesis + 7 days to physiological maturity (g m^-2^), IPARacc_E40-InB: IPAR accumulated from emergence + 40 days to initiation of booting , IPARacc InB-A7: IPAR accumulated from initiation of booting to anthesis + 7 days, IPARacc_A7-PM: IPAR accumulated from anthesis + 7 days to physiological maturity, IPARacc_E40-PM: IPAR accumulated from emergence + 40 days to physiological maturity.

**Supplementary Table 6.** Phenotypic correlations between fractional light interception (FLI) and above-ground biomass at different growth stages for 12 spring CIMMYT wheat genotypes. Values based on means from the combined analysis in 2018-19 and 2019-20 in raised beds (B) and flat basins (F).

|  | RAISED BEDS (B) | | | FLAT BASINS (F) | | |
| --- | --- | --- | --- | --- | --- | --- |
|  | FLIE40 | FLIInB | FLIA7 | FLIE40 | FLIInB | FLIA7 |
| BME40 | -0.07 | -0.32 | -0.19 | -0.20 | 0.56† | -0.02 |
| BMInB | **0.59*** | **0.71*** | 0.50† | 0.39 | 0.48 | 0.07 |
| BMA7 | **0.73**** | **0.72**** | **0.71**** | 0.27 | 0.45 | -0.21 |
| BMPM | **0.70*** | **0.82**** | **0.84***** | 0.14 | 0.31 | -0.01 |

BME40: biomass at emergence + 40 days (g m^-2^), BMInB: biomass at initiation of booting (g m^-2^), BMA7: biomass at anthesis + 7 days (g m^-2^), BMPM: biomass at physiological maturity (g m^-2^), FLIE40: light interception at emergence + 40 days, FLIInB: light interception at initiation of booting, FLIA7: light interception at anthesis + 7 days. *P < 0.05, **P < 0.01, ***P < 0.001, †P < 0.10.

**Supplementary Table 7.** Broad-sense heritability (H^2^) for yield, yield components, biomass at maturity, phenology expressed in days after emergence (DAE), number of shoots from the three combined analysis across 2017-18, 2018-19 and 2019-20 and RUE from the two combined analysis (2018-19 and 2019-20). Plants (m^-2^)‡ (data 2019-20).

| Trait | H^2^ |
| --- | --- |
| YLD (g m^-2^) | 0.90 |
| TGW (g) | 0.95 |
| HI | 0.94 |
| GM2 (m^-2^) | 0.97 |
| SM2 (m^-2^) | 0.87 |
| GPS | 0.73 |
| BMPM (g m^-2^) | 0.86 |
| HeightPM (cm) | 0.82 |
| ShootsE40 (m^-2^) | 0.17 |
| ShootsA7 (m^-2^) | 0.63 |
| DTInB (DAE) | 0.75 |
| DTH (DAE) | 0.46 |
| DTA (DAE) | 0.45 |
| DTPM (DAE) | 0.28 |
| Plants (m^-2^)‡ | 0.86 |
| RUE_E40InB | 0.42 |
| RUE_InBA7 | 0.71 |
| RUE_GF | 0.09 |
| RUE_preGF | 0.82 |
| RUET | 0.84 |

YLD: grain yield, TGW: thousand grain weight, HI: harvest index, GM2: grain number per square meter, SM2: spikes per square meter, GPS: grains per spike, BMPM: biomass at physiological maturity, HeightPM: plant height at physiological maturity, ShootsE40: fertile shoots at emergence + 40 days, ShootsA7: fertile shoots at seven days to anthesis, DTInB (DAE, days after emergence): days to initiation of booting (GS41), DTH (DAE): days to heading (GS55), DTA (DAE): days to anthesis (GS65), DTPM (DAE): days to physiological maturity (GS87), Plants: number of plants per square meter, RUE_E40InB: RUE calculated from forty days after emergence to initiation of booting, RUE_InBA7: RUE calculated from initiation of booting to seven days after emergence, RUE_GF: RUE calculated during the grain filling period from seven days after anthesis to physiological maturity, RUE_preGF: RUE pre grain-filling from forty days after emergence to seven days after anthesis, RUET: RUET total, from forty days after emergence to physiological maturity (RUET).
